# GPR183 senses 7α,25-OHC to drive T_reg_ differentiation and tumor immune evasion via AXL-JAK2-STAT5 signaling

**DOI:** 10.1126/sciadv.aeh9736

**Published:** 2026-10-30

**Authors:** Yang Qu, Yihan Xu, Jingchao Bai, Yuhan Zhang, Ning Ji, Xinxin Li, Hui Li

**Affiliations:** ^1^ Department of Gastrointestinal Cancer Biology, Tianjin Medical University Cancer Institute and Hospital, Tianjin, China.; ^2^ Tianjin Key Laboratory of Digestive Cancer, Tianjin Clinical Research Center for Cancer, Tianjin, China.; ^3^ National Clinical Research Center for Cancer, Tianjin Medical University Cancer Institute and Hospital, Tianjin, China.; ^4^ Department of Gynecologic Oncology, Tianjin Medical University Cancer Institute and Hospital, Tianjin, China.; ^5^ Department of Radiation Oncology, Tianjin’s Clinical Research Center for Cancer, Key Laboratory of Cancer Prevention and Therapy, Tianjin Medical University Cancer Institute and Hospital, Tianjin, China.

## Abstract

Regulatory T cell (T_reg_) accumulation within the tumor microenvironment is a major contributor to immune evasion and immunotherapy resistance. However, the metabolic signals orchestrating T_reg_ lineage commitment remain poorly defined. We identify that the oxysterol 7α,25-dihydroxycholesterol (7α,25-OHC), produced by Vimentin^+^ stromal cells, acts through GPR183 to promote T_reg_ differentiation and suppressive identity. In patients with gastric cancer (GC), GPR183^+^ T_reg_ infiltration correlates with advanced disease and poor survival. In syngeneic tumor models, conditional GPR183 deletion, specifically in CD4^+^ T cells or T_reg_ cells, demonstrates its cell-intrinsic requirement for high-cholesterol-diet–induced T_reg_ expansion and tumor growth, thereby abrogating immune evasion and restoring CD8^+^ T cell effector function. Mechanistically, 7α,25-OHC–GPR183 signaling activates the AXL-JAK2-STAT5 pathway to reinforce Foxp3 expression. Last, GPR183 antagonism modestly enhances anti-PD-1 efficacy in a model-dependent manner and prevents therapy-induced T_reg_ expansion. These findings identify the 7α,25-OHC–GPR183–AXL axis as a potential metabolic checkpoint linking cholesterol metabolism to immune suppression, suggesting a potential therapeutic strategy to enhance antitumor immunity.

## INTRODUCTION

Gastric cancer (GC) remains a leading cause of cancer-related mortality worldwide, characterized by a profoundly immunosuppressive tumor microenvironment (TME) that limits the efficacy of immune checkpoint inhibitors (ICIs) (*1*–*3*). Accumulation of regulatory T cells (T_reg_ cells) within the TME is a cardinal feature of immune evasion in GC and a key determinant of poor prognosis and resistance to anti-PD-1/PD-L1 therapy (*2*, *4*–*10*). Despite the clinical importance of T_reg_ cells, the specific environmental cues within the GC microenvironment that drive their differentiation, stability, and suppressive function remain incompletely understood.

Emerging evidence points to a pivotal role of metabolic reprogramming in shaping immune cell composition and function within tumors (*11*, *12*). Cholesterol metabolism, in particular, has garnered substantial attention, as its dysregulation in the TME can influence the fate and activity of both effector and regulatory immune cells (*13*, *14*). T_reg_ cells are known to undergo metabolic adaptation to sustain their suppressive identity (*15*, *16*), yet the molecular links connecting specific cholesterol-derived metabolites to T_reg_ lineage commitment and functional stability are largely undefined. Oxysterols, oxygenated derivatives of cholesterol, have recently emerged as key immunometabolic regulators that can modulate immune cell behavior through interactions with nuclear receptors, such as liver X receptors (LXRs) and retinoic acid receptor–related orphan receptor γt (RORγt), or G protein–coupled receptors (GPCRs) (*17*–*19*).

Among these, 7α,25-dihydroxycholesterol (7α,25-OHC) serves as the high-affinity ligand for GPR183 (also known as EBI2), a GPCR expressed on various immune cells, including B cells, T cells, and innate lymphoid cells (*20*–*23*). While GPR183 is known to guide lymphocyte migration within secondary lymphoid organs (*21*, *24*, *25*), its role in directly regulating T cell differentiation, particularly in the context of a cholesterol-rich TME, has not been explored. A critical knowledge gap exists regarding whether and how the 7α,25-OHC–GPR183 axis might actively orchestrate T_reg_ biology and contribute to the immunosuppressive landscape of solid tumors like GC.

In this study, we hypothesize that stromal-derived 7α,25-OHC acts as a metabolic instruction signal, engaging GPR183 on CD4^+^ T cells to drive T_reg_ differentiation and fortify their suppressive function, thereby promoting immune evasion and resistance to immunotherapy with potential relevance to GC. We provide evidence establishing this axis as a critical link between cholesterol metabolism and T_reg_-mediated immune suppression, and demonstrate that targeting GPR183 represents a promising strategy to overcome metabolic barriers and enhance the efficacy of PD-1 blockade.

## RESULTS

### The 7α,25-OHC/GPR183 axis is up-regulated in the stroma and correlates with T_reg_ accumulation and poor prognosis in GC

To elucidate how cholesterol metabolites and their cognate receptors modulate the heterogeneous immune microenvironment in GC, we focused on the 7α,25-OHC/GPR183 signaling axis. Because the coexpression of the key oxysterol-synthesizing enzymes CH25H and CYP7B1 serves as a reliable surrogate for local 7α,25-OHC production (*20*, *26*, *27*), we analyzed the single-cell RNA sequencing (RNA-seq) dataset GSE134520 using TISCH and found that *CH25H* and *CYP7B1* were preferentially expressed in fibroblasts and myofibroblasts (fig. S1, A to F). This expression pattern suggests that stromal cells are the primary source of 7α,25-OHC in the TME. This finding aligns with published studies identifying fibroblasts as the principal producers of this oxysterol in other pathological settings (*20*, *26*). To directly confirm the presence of 7α,25-OHC in human GC tissues, we performed liquid chromatography–tandem mass spectrometry (LC-MS/MS) on freshly frozen tumor specimens from five treatment-naïve patients. 7α,25-OHC was detectable in four out of five tumors, with concentrations ranging from 0.68 to 1.85 ng/g of tissue (fig. S1G). To validate these observations in clinical specimens and examine the spatial context of 7α,25-OHC synthesis, we characterized the distribution and cellular sources of CH25H and CYP7B1 in GC tissues. We used multiplex immunofluorescence (mIF) staining on a cohort of 93 GC patient tissues (table S1). The results revealed that CH25H and CYP7B1 were predominantly colocalized within Vimentin^+^ stromal cells (Fig. 1A). Quantitative analysis showed that the frequency of CH25H^+^CYP7B1^+^ cells within the Vimentin^+^ stromal cells was significantly elevated in advanced-stage (III–IV) tumors compared to early-stage (I–II) cases (Fig. 1B; *P* < 0.0001). However, the infiltration levels of these enzyme-expressing Vimentin^+^ stromal cells alone did not significantly correlate with overall survival (OS) (Fig. 1C; *P* = 0.4301).

**Fig. 1.**
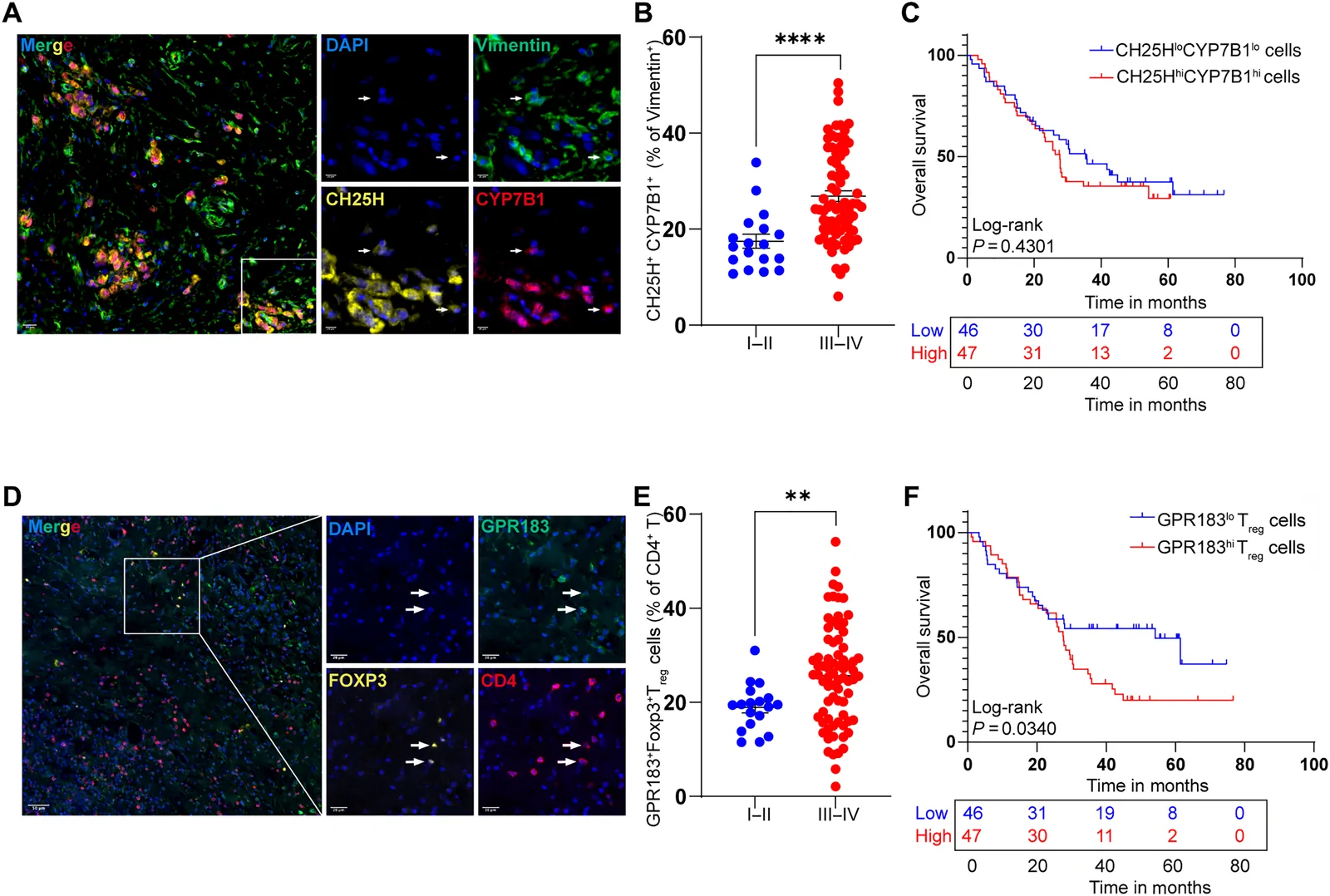
Stromal cell–derived 7α,25-OHC–synthesizing enzymes and GPR183^+^ T_reg_ infiltration correlate with GC progression. (**A**) Representative mIF images showing the colocalization of 7α,25-OHC synthetic enzymes (CH25H and CYP7B1) with Vimentin^+^ stromal cells in GC tissues from 93 independent patients. Scale bars, 50 μm in merged images and 20 μm in magnified single-channel inserts. (**B**) Percentage of CH25H^+^CYP7B1^+^ cells among Vimentin^+^ stromal cells in early-stage (I–II, *n* = 18) versus advanced-stage (III–IV, *n* = 75) tumors. Data are presented as means ± standard deviation. Statistical significance was determined by Mann-Whitney test. (**C**) Kaplan-Meier survival analysis of OS based on the infiltration levels of CH25H^+^CYP7B1^+^ stromal cells (patients stratified by median value; log-rank test, *P* = 0.4301). (**D**) Representative mIF images showing the infiltration of GPR183^+^Foxp3^+^ cells among CD4^+^ T cells in gastric adenocarcinoma tissues (*n* = 93). Scale bars, 50 μm in merged images and 20 μm in magnified single-channel inserts. (**E**) Quantitative comparison of GPR183^+^ T_reg_ proportion between early-stage (I–II, *n* = 18) and advanced-stage (III–IV, *n* = 75) patients. Data are presented as means ± standard deviation. Statistical significance was determined by Mann-Whitney test. (**F**) Kaplan-Meier survival curves for OS in patients with GC stratified by GPR183^+^ T_reg_ infiltration (patients stratified by median value; log-rank test, *P* = 0.0340). **P < 0.01; ****P < 0.0001.

Given that the oxysterol 7α,25-OHC is a bioactive lipid capable of modulating immune cell phenotypes, we further investigated the clinical relevance of GPR183 expression in T_reg_ cells (table S2). mIF analysis revealed the presence of GPR183^+^Foxp3^+^ T_reg_ cells in the CD4^+^ T cell compartment (Fig. 1D). Notably, the proportion of GPR183^+^Foxp3^+^ T_reg_ cells among tumor-infiltrating CD4^+^ T cells was significantly higher in patients with advanced-stage GC (Fig. 1E; *P* = 0.0045) and was significantly associated with shortened OS (Fig. 1F; *P* = 0.0340). Collectively, these findings reveal a dichotomy in the 7α,25-OHC/GPR183 axis: While ligand-producing Vimentin^+^ stromal cells associate with tumor progression, GPR183^+^ T_reg_ infiltration is substantially associated with poor survival in GC.

### GPR183 in CD4^+^ T cells is essential for cholesterol-driven tumor growth and T_reg_-mediated immunosuppression

To assess the role of GPR183 in modulating tumor progression under metabolic stress, we challenged mice with conditional deletion of GPR183 in CD4^+^ T cells (*CD4*^cre^*Gpr183*^fl/fl^, hereafter referred to as *Gpr183*^Δ*CD4*^) and their control littermates [*Gpr183*^fl/fl^, hereafter referred to as wild type (WT)] with MC38 or B16F10 tumors under either a standard diet (SD) or a 2% high-cholesterol diet (HCD) (Fig. 2A). Tumors from the HCD group exhibited significantly up-regulated expression of CH25H and CYP7B1, confirming enriched local production of 7α,25-OHC within the TME (Fig. 2B). Consistently, LC-MS/MS analysis of tumor tissues confirmed that HCD feeding significantly increased intratumoral 7α,25-OHC levels compared with SD controls, further demonstrating that dietary cholesterol enhances local accumulation of this GPR183 ligand within the TME (Fig. 2C). In WT mice, HCD consumption significantly accelerated MC38 tumor growth. This HCD-promoted progression was largely neutralized in *Gpr183*^Δ*CD4*^ mice; these mice displayed significantly reduced tumor volumes and demonstrated relative resistance to the tumor-promoting effects of dietary cholesterol (Fig. 2D). Consistent with the tumor kinetics, HCD induced a robust increase in the frequency and Foxp3 mean fluorescence intensity (MFI) of tumor-infiltrating T_reg_ cells in WT mice, whereas this induction was absent in *Gpr183*^Δ*CD4*^ mice (Fig. 2E). Furthermore, the expression of PD-1 on intratumoral T_reg_ cells was significantly reduced in *Gpr183*^Δ*CD4*^ mice, suggesting a less suppressive T_reg_ phenotype (Fig. 2F). In contrast, the levels of CTLA-4, interleukin-10 (IL-10), and LAP were not significantly affected (fig. S2, A to C), indicating that GPR183 selectively tunes the T_reg_ suppressive landscape under metabolic stress. This reduction in T_reg_ accumulation was accompanied by enhanced antitumor effector responses. Compared with WT controls, *Gpr183*^Δ*CD4*^ mice exhibited increased intratumoral CD8^+^ T cell infiltration, a higher CD8^+^ T cell/T_reg_ ratio, and enhanced production of interferon-γ (IFN-γ) and Granzyme B (GzmB), particularly under HCD conditions (Fig. 2, G to J). Control experiments confirmed that these observations were not due to developmental defects, as systemic T cell populations remained comparable under steady-state conditions (fig. S2, D and E). Consistently, HCD did not significantly affect the accumulation of M2-like macrophages or myeloid-derived suppressor cells (MDSCs) in the TME (fig. S2, F and G), suggesting that GPR183 expression in CD4^+^ T cells, rather than global myeloid changes, drives the observed immune evasion.

**Fig. 2.**
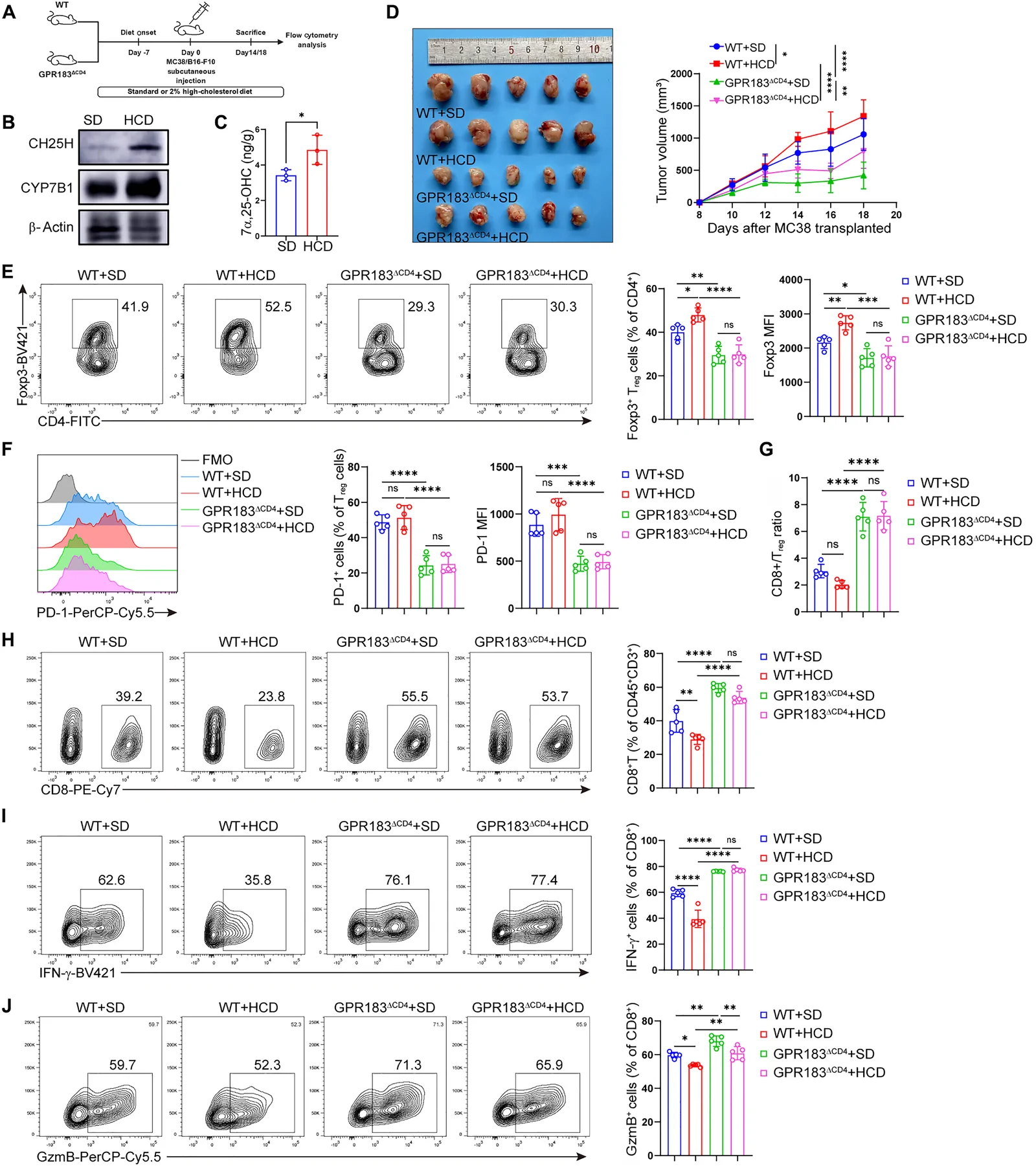
T cell–intrinsic GPR183 mediates cholesterol-driven T_reg_ accumulation and immune suppression in tumors. (**A**) Schematic representation of the experimental workflow. Created in BioRender. A. Qu (2026); https://BioRender.com/6ke2w7y. WT and *Gpr183*^Δ*CD4*^ mice were fed SD or HCD and challenged with MC38 or B16F10 tumors. (**B**) Representative Western blot analysis of CH25H and CYP7B1 in MC38 tumors from SD- or HCD-fed mice. β-Actin served as a loading control; *n* = 3 biological replicates (mice) per group. (**C**) Quantification of 7α,25-OHC levels in MC38 tumor tissues from mice fed with SD or HCD was performed by LC-MS/MS analysis. Data are presented as means ± standard deviation (*n* = 3 mice per group). Statistical significance was determined by two-tailed unpaired Student’s *t* test. *P < 0.05. (**D**) Representative images of dissected tumors (left) and tumor growth curves (right) of MC38 tumors in the indicated groups; *n* = 5 biological replicates (mice) per group. (**E**) Representative flow cytometry plots (left) and statistical quantification (right) of Foxp3^+^ T_reg_ frequencies and Foxp3 MFI. (**F**) Statistical analysis of PD-1 MFI on intratumoral T_reg_ cells. (**G**) Intratumoral CD8^+^ T cell/T_reg_ ratio in WT and *Gpr183*^Δ*CD4*^ mice under SD or HCD conditions. (**H**) Representative flow plots and quantification of intratumoral CD8^+^ T cell frequencies. (**I** and **J**) Representative flow plots and quantification of IFN-γ^+^ (I) and GzmB^+^ (J) CD8^+^ T cells. Data in (D) to (J) are presented as means ± standard deviation (*n* = 5 biological replicates, mice per group). Statistical significance for tumor growth curves in (D) was determined by two-way repeated-measures ANOVA followed by Tukey’s post hoc test. Statistical significance for endpoint comparisons in (D) to (J) was determined by two-way ANOVA followed by Tukey’s post hoc test for multiple comparisons. *P < 0.05; **P < 0.01; ***P < 0.001; ****P < 0.0001; ns, not significant.

The essential role of GPR183 was further recapitulated in the B16F10 melanoma model, albeit with expected variations due to its lower immunogenicity. Similar to the MC38 model, *Gpr183*^Δ*CD4*^ mice were refractory to HCD-induced acceleration of B16F10 growth and exhibited a significant reduction in T_reg_ infiltration (fig. S3, A and B). Correspondingly, while HCD showed a modest trend toward reducing CD8^+^ T cell density, the ablation of GPR183 shifted the effector landscape by enhancing CD8^+^ T cell infiltration and GzmB levels, despite less pronounced changes in markers such as PD-1 and IFN-γ (fig. S3, C to F). These findings, particularly the restoration of the effector-to-suppressor ratio in *Gpr183*^Δ*CD4*^ mice, largely offset the potential suppressive effects of dietary cholesterol and support a broader role for GPR183 in cholesterol-induced, T_reg_-mediated immune escape across distinct tumor models.

### 7α,25-OHC directly promotes T_reg_ differentiation via GPR183 in vitro and in an adoptive-transfer setting

To further elucidate the cellular mechanisms underlying the reduced T_reg_ accumulation observed in *Gpr183*^Δ*CD4*^ tumor-bearing mice, we investigated whether GPR183 activation directly modulates T_reg_ differentiation. Naïve CD4^+^ T cells isolated from human peripheral blood mononuclear cells (PBMCs) were cultured under low-dose T_reg_-polarizing conditions to sensitively detect GPR183-mediated effects. Compared with controls, 7α,25-OHC treatment markedly potentiated T_reg_ differentiation, an effect that was completely abrogated by the GPR183-specific antagonist NIBR189 (Fig. 3A). Cell viability remained unaffected by these treatments, confirming that the observed changes were not due to cytotoxicity (fig. S4A). These findings were recapitulated in murine naïve CD4^+^ T cells, highlighting the evolutionary conservation of the GPR183 pathway (Fig. 3B). To genetically validate these observations, we compared the polarizing potential of WT and *Gpr183*^Δ*CD4*^ naïve CD4^+^ T cells. Flow cytometry revealed that while 7α,25-OHC significantly enhanced T_reg_ induction and Foxp3 MFI in WT cells, GPR183 deficiency rendered the cells refractory to ligand-induced differentiation (Fig. 3C). In line with the increased Foxp3 expression, 7α,25-OHC significantly up-regulated the functional marker PD-1 on induced T_reg_ cells—an effect consistently abolished by either pharmacological inhibition or genetic ablation of GPR183 (Fig. 3, D and E). These results suggest that the 7α,25-OHC/GPR183 axis promotes not only the differentiation but also the activation of T_reg_ cells.

**Fig. 3.**
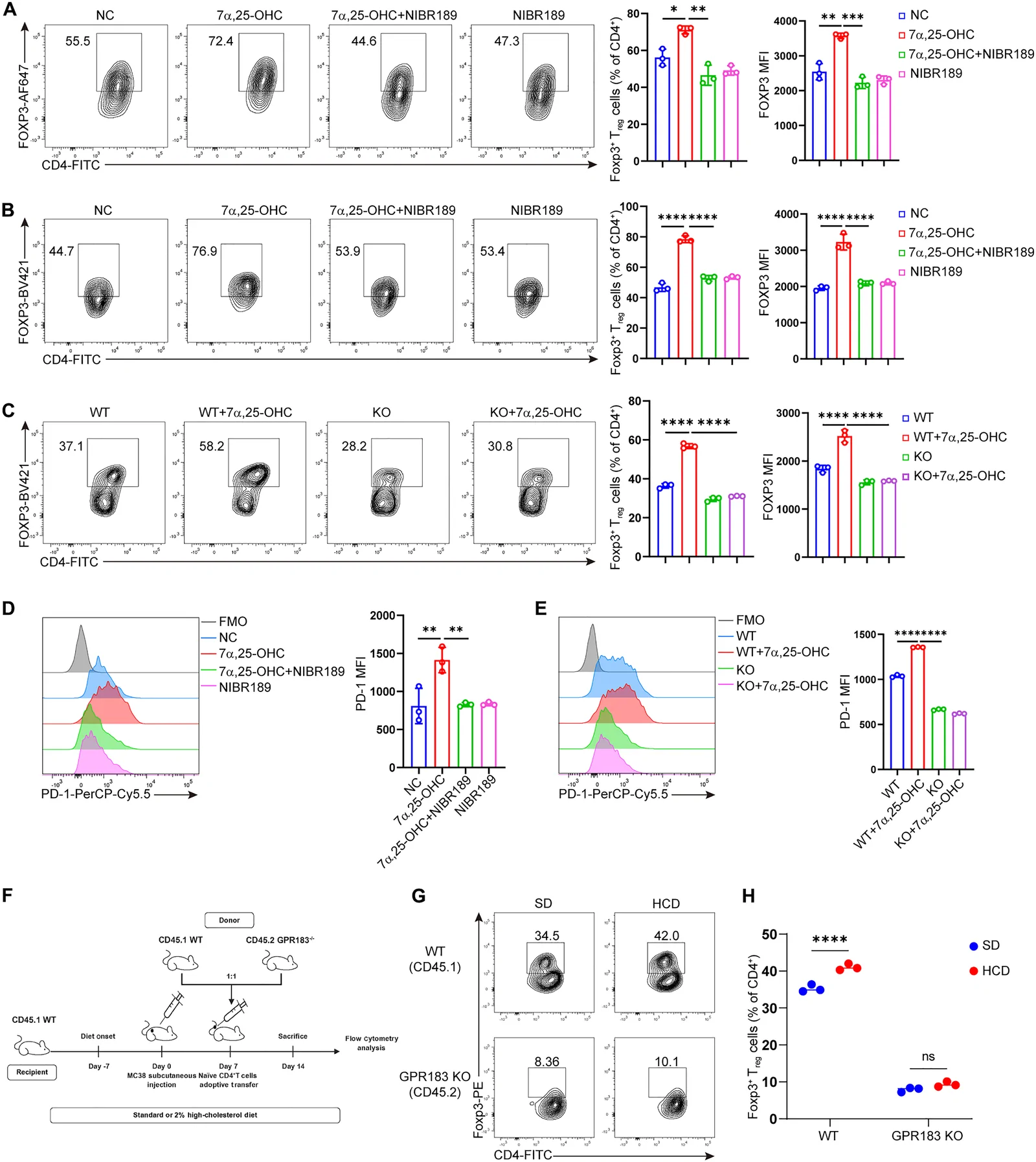
Activation of the 7α,25-OHC–GPR183 axis promotes T_reg_ differentiation and Foxp3 expression in vitro and in an adoptive-transfer setting. (**A** and **B**) Flow cytometry analysis and quantification of T_reg_ differentiation from (A) human (*n* = 3 biological replicates from independent donors) and (B) murine (*n* = 3 biological replicates, mice) naïve CD4^+^ T cells treated with 7α,25-OHC (100 nM) in the presence or absence of the GPR183 antagonist NIBR189 (250 nM). (**C**) Representative flow cytometry plots and percentage of Foxp3^+^ T_reg_ cells differentiated from WT and *Gpr183*^−/−^ murine naïve CD4^+^ T cells following 7α,25-OHC stimulation. (**D** and **E**) Representative histograms and quantitative analysis of PD-1 expression in T_reg_ cells under the indicated treatment conditions or genotypes. Data in (C) to (E) are presented as means ± standard deviation (*n* = 3 biological replicates). (**F**) Experimental schema of the competitive adoptive transfer model. Created in BioRender. A. Qu (2026); https://BioRender.com/tz5eurv. A 1:1 mixture of WT (CD45.1^+^) and *Gpr183*^−/−^ (CD45.2^+^) naïve CD4^+^ T cells was intravenously injected into MC38 tumor-bearing mice fed with either SD or HCD. (**G**) Representative flow cytometry plots showing the frequency of tumor-infiltrating T_reg_ cells derived from WT and *Gpr183*^−/−^ donor cells in the SD and HCD groups. (**H**) Statistical quantification of the proportion of Foxp3^+^ T_reg_ cells among the transferred CD45.1^+^ (WT) and CD45.2^+^ (*Gpr183*^−/−^) populations. Data in (F) to (H) are presented as means ± standard deviation (*n* = 5 biological replicates, mice per group). *P < 0.05; **P < 0.01; ***P < 0.001; ****P < 0.0001; ns, not significant.

To evaluate the in vivo relevance of the 7α,25-OHC/GPR183 axis, we used a competitive adoptive transfer model. Recipient CD45.1 mice were first preconditioned with either an SD or an HCD for 1 week prior to subcutaneous MC38 tumor transplantation. One week posttransplantation, a 1:1 mixture of WT (CD45.1^+^) and *Gpr183*^−/−^ (CD45.2^+^) naïve CD4^+^ T cells was intravenously injected into these tumor-bearing hosts. Seven days following the adoptive transfer, tumor-infiltrating lymphocytes (TILs) were harvested for flow cytometry analysis using a comprehensive gating strategy (Fig. 3F and fig. S4B). The analysis revealed that HCD notably promoted the conversion of WT naïve CD4^+^ T cells into T_reg_ cells (Fig. 3, G and H). Conversely, GPR183 deficiency significantly impaired this differentiation process within the same host, even under ligand-enriched conditions (Fig. 3, G and H). Collectively, these results demonstrate that the 7α,25-OHC–GPR183 axis plays a critical role in promoting T_reg_ differentiation both in vitro and in the adoptive-transfer setting.

### GPR183 is critical for maintaining the suppressive identity and functional stability of mature T_reg_ cells

To determine whether GPR183 is required for the maintenance of T_reg_ suppressive function postdifferentiation, we first performed an in vitro suppression assay. While WT T_reg_ cells effectively inhibited the proliferation of CD8^+^ T cells, *Gpr183*^−/−^ T_reg_ cells exhibited a significantly compromised suppressive capacity (Fig. 4A), indicating a functional defect inherent to GPR183-deficient T_reg_ cells.

**Fig. 4.**
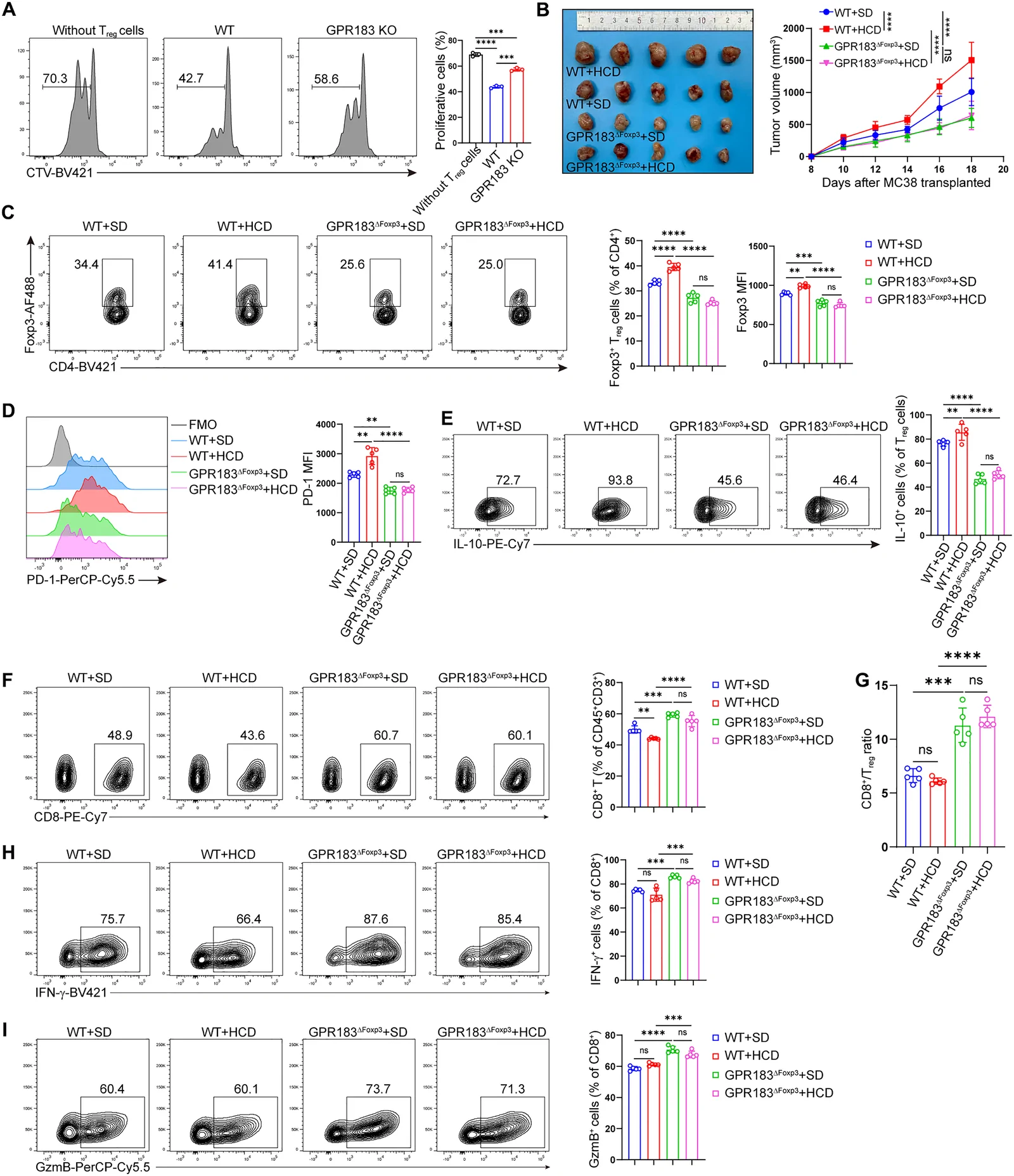
GPR183 is indispensable for maintaining the suppressive identity and functional stability of mature T_reg_ cells. (**A**) In vitro suppression assay showing the proliferative capacity of CellTrace Violet (CTV)–labeled CD8^+^ T cells cultured alone (without T_reg_ cells) or with differentiated WT or *Gpr183*^−/−^ T_reg_ cells. Representative histograms (left) and statistical quantification of proliferative cells (right) are shown. Data are presented as means ± standard deviation (*n* = 3 biological replicates). Statistical significance was determined by one-way ANOVA followed by Tukey’s post hoc test. (**B**) MC38 tumor growth curves (right) and representative tumor images (left) in WT and *Gpr183*^Δ*Foxp3*^ mice fed SD or HCD; *n* = 5 biological replicates (mice) per group. (**C**) Representative flow cytometry plots and statistical analysis of intratumoral T_reg_ frequencies and Foxp3 MFI in the indicated groups. (**D** and **E**) Representative flow cytometry histograms or plots and MFI or frequency quantification of suppressive markers PD-1 (D) and IL-10 (E) in intratumoral T_reg_ cells. (**F**) Representative flow cytometry plots and frequencies of intratumoral CD8^+^ T cells among the four groups. (**G**) Intratumoral CD8^+^ T cell/T_reg_ ratio in WT and *Gpr183*^Δ*Foxp3*^ mice under SD or HCD conditions. (**H** and **I**) Representative flow cytometry plots and quantification of effector molecules IFN-γ (H) and GzmB (I) expression in intratumoral CD8^+^ T cells. Data in (B) to (I) are presented as means ± standard deviation (*n* = 5 biological replicates, mice per group). Statistical significance was determined by two-way ANOVA followed by Tukey’s post hoc test. *P < 0.05; **P < 0.01; ***P < 0.001; ****P < 0.0001; ns, not significant.

We next used control littermates (*Gpr183*^fl/fl^, hereafter WT) and *Gpr183*^fl/fl^*Foxp3*^cre^ (hereafter *Gpr183*^Δ*Foxp3*^) mice to specifically ablate GPR183 in mature T_reg_ cells. Consistent with our findings in the *Gpr183*^Δ*CD4*^ model, HCD consumption accelerated tumor progression in WT mice, whereas this effect was completely abolished in *Gpr183*^Δ*Foxp3*^ mice across both MC38 and B16F10 models (Fig. 4B and fig. S5, A and B). Notably, GPR183 deficiency in mature T_reg_ cells resulted in a marked reduction in both T_reg_ infiltration and Foxp3 expression levels, regardless of dietary cholesterol (Fig. 4C). This phenomenon was particularly pronounced in the B16F10 melanoma model, where HCD-induced T_reg_ accumulation was more drastically curtailed in *Gpr183*^Δ*Foxp3*^ mice (fig. S5C).

Further characterization revealed that GPR183 ablation consistently down-regulated key suppressive markers, including PD-1 and IL-10, in intratumoral T_reg_ cells across both MC38 and B16F10 models (Fig. 4, D and E, and fig. S5, D and E). This reduction effectively abrogated the HCD-induced elevation of the T_reg_ suppressive identity, maintaining these markers at levels comparable to or lower than those of WT counterparts.

Consistent with the impaired T_reg_ stability, the loss of GPR183 in T_reg_ cells led to a profound enhancement of CD8^+^ T cell effector functions across both models. Regardless of the dietary cholesterol intake, *Gpr183*^Δ*Foxp3*^ mice exhibited a significant increase in the intratumoral CD8^+^ T cell population (Fig. 4F and fig. S5F). This was accompanied by a markedly elevated CD8^+^ T cell/T_reg_ ratio, indicating a shift toward a more favorable antitumor immune balance within the TME (Fig. 4G). Furthermore, CD8^+^ T cells from *Gpr183*^Δ*Foxp3*^ tumors displayed significantly increased production of IFN-γ and GzmB compared with their WT counterparts (Fig. 4, H and I, and fig. S5, G and H). These data suggest that GPR183-deficient T_reg_ cells are less capable of restraining effector T cell recruitment and activation, thereby shifting the TME toward a proinflammatory state.

To further determine the mechanisms underlying the reduced intratumoral T_reg_ accumulation following GPR183 deletion, we assessed whether altered trafficking, retention, survival, or local proliferation contributed to this phenotype. Given the established role of GPR183 in immune-cell positioning, we first examined lymphoid homing and tissue-retention programs. CCR7 and CD62L expression was largely comparable among genotypes in tumor-draining lymph nodes, while only a modest reduction was observed in tumor-infiltrating *Gpr183*^Δ*Foxp3*^ T_reg_ cells, which was not observed in *Gpr183*^Δ*CD4*^ T_reg_ cells (fig. S6A). These findings argue against a major defect in T_reg_ recruitment as the primary cause of reduced tumor infiltration. Similarly, expression of the tissue-residency markers CD69 and CD103 remained comparable across groups (fig. S6B), suggesting that impaired retention does not account for the diminished intratumoral T_reg_ compartment. We next examined whether altered local proliferation or survival contributed to the reduced intratumoral T_reg_ accumulation following GPR183 deletion. Ki-67 expression was significantly reduced in *Gpr183*^Δ*CD4*^ T_reg_ cells and showed a similar downward trend in *Gpr183*^Δ*Foxp3*^ T_reg_ cells (fig. S6C), suggesting that impaired local proliferative capacity may contribute to the diminished accumulation of GPR183-deficient T_reg_ cells within tumors. In contrast, cleaved caspase-3 expression remained comparable among all groups (fig. S6D), indicating that increased apoptosis is unlikely to account for the reduction in the intratumoral T_reg_ compartment.

Collectively, these data demonstrate that GPR183 not only sustains T_reg_ suppressive identity and functional stability but also supports their maintenance within the TME, thereby reinforcing immune suppression and tumor progression.

### GPR183 signals through the AXL-JAK2-STAT5 axis to contribute to T_reg_ differentiation

To elucidate the downstream molecular mechanisms by which 7α,25-OHC–GPR183 signaling drives T_reg_ differentiation, we performed transcriptomic profiling on in vitro–differentiated T_reg_ cells from WT and *Gpr183*^−/−^ mice. Among the differentially expressed genes (DEGs), the receptor tyrosine kinase (RTK) AXL was identified as a key candidate, being markedly down-regulated in GPR183-deficient cells (Fig. 5A). We further validated this finding at the protein level; while 7α,25-OHC treatment up-regulated AXL expression in WT CD4^+^ T cells, this induction was largely abrogated by the genetic ablation of *Gpr183* (Fig. 5B).

**Fig. 5.**
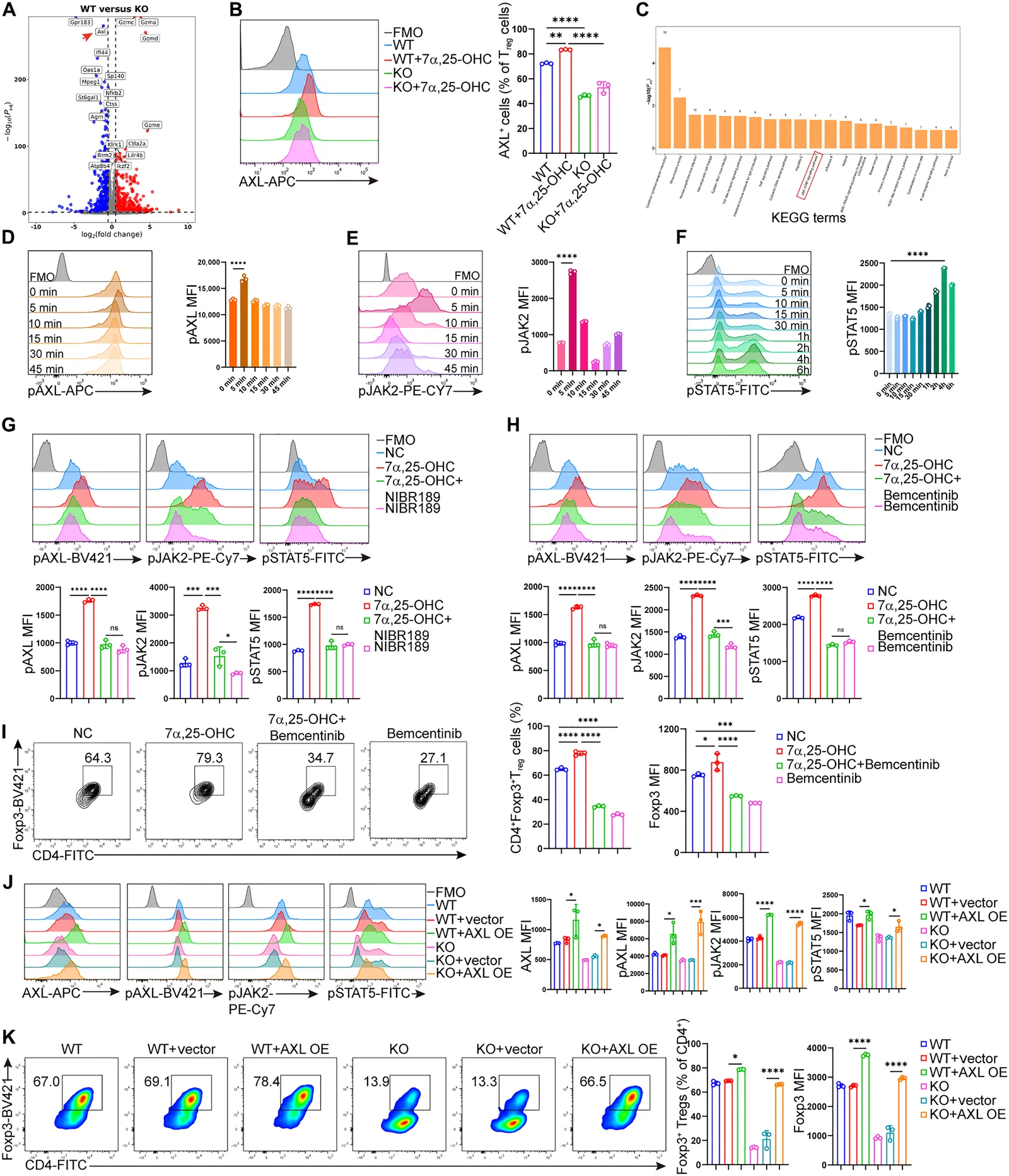
GPR183-mediated AXL activation contributes to T_reg_ differentiation through the JAK2-STAT5 signaling axis. (**A**) Volcano plot identifying DEGs between in vitro–differentiated WT and *Gpr183*^−/−^ T_reg_ cells. The arrow highlights AXL as a markedly down-regulated candidate in GPR183-deficient cells; *n* = 5 biological replicates per genotype. (**B**) Representative flow cytometry histograms (left) and quantification (right) of AXL^+^ T_reg_ cells from WT and *Gpr183*^−/−^ mice treated with or without 7α,25-OHC. (**C**) KEGG pathway enrichment analysis of DEGs, with the JAK-STAT signaling pathway indicated by the red box. (**D** to **F**) Phosflow analysis of phosphorylation kinetics for AXL (D), JAK2 (E), and STAT5 (F) in murine naïve CD4^+^ T cells following 7α,25-OHC stimulation. Representative histograms and MFI are shown. (**G** and **H**) Phosflow analysis of pAXL, pJAK2, and pSTAT5 levels in murine naïve CD4^+^ T cells treated with 7α,25-OHC in the presence or absence of NIBR189 (G) or Bemcentinib (H). (**I**) Representative flow cytometry plots and quantification of CD4^+^Foxp3^+^ T_reg_ frequencies and Foxp3 MFI following treatment with 7α,25-OHC and/or Bemcentinib. (**J**) Phosflow analysis of AXL, pAXL, pJAK2, and pSTAT5 levels in WT and *Gpr183*^−/−^ CD4^+^ T cells following retroviral transduction with empty vector or AXL-overexpression construct. (**K**) Quantification of T_reg_ differentiation and Foxp3 MFI after AXL rescue. Data in (B) and (D) to (K) are presented as means ± standard deviation (*n* = 3 biological replicates from three independent experiments). Statistical significance was determined by one-way or two-way ANOVA followed by Tukey’s post hoc test, as appropriate. *P < 0.05; **P < 0.01; ***P < 0.001; ****P < 0.0001.

Informed by our Kyoto Encyclopedia of Genes and Genomes (KEGG) enrichment analysis, which highlighted the Janus kinase–signal transducer and activator of transcription (JAK-STAT) signaling pathway, and prior evidence linking AXL to the JAK-STAT5 axis (*27*, *28*), we investigated whether this cascade operates downstream of GPR183 (Fig. 5C). To capture the priming events of this pathway, we analyzed the phosphorylation kinetics in isolated naïve CD4^+^ T cells shortly after 7α,25-OHC stimulation. Phosflow analysis revealed that 7α,25-OHC triggered rapid phosphorylation of AXL and JAK2, both peaking within 5 min, followed by a sustained increase in STAT5 phosphorylation that peaked at 4 hours (Fig. 5, D to F). The requirement for the GPR183-AXL module in activating this cascade was confirmed via targeted pharmacological inhibition. Reflecting the inhibitory effects on T_reg_ differentiation observed earlier (Fig. 3B), the GPR183 antagonist NIBR189 significantly attenuated the 7α,25-OHC–induced phosphorylation of AXL, as well as the downstream activation of JAK2 and STAT5 (Fig. 5G). Similarly, treatment with the AXL-specific inhibitor Bemcentinib not only abrogated AXL activation but also subsequently suppressed the phosphorylation of JAK2 and STAT5 (Fig. 5H). Crucially, consistent with the signaling blockade, Bemcentinib significantly inhibited 7α,25-OHC–induced T_reg_ differentiation and Foxp3 expression (Fig. 5I), further reinforcing the requirement of the GPR183-AXL axis in this process. These findings support that GPR183 acts as the upstream trigger that contributes to the AXL-dependent signaling cascade. Mechanistically, as GPCRs frequently transactivate RTKs via non–receptor tyrosine kinases, we examined the role of Src family kinases (SFKs) in this cross-talk (*29*, *30*). Phosflow analysis revealed that 7α,25-OHC triggered a rapid, GPR183-dependent phosphorylation of Src (fig. S7A). Furthermore, pharmacological inhibition of SFKs by PP2 completely abolished the 7α,25-OHC–induced phosphorylation of AXL, JAK2, and STAT5 (fig. S7B), identifying SFKs as the critical biochemical link between GPR183 and the AXL axis.

Last, we performed genetic rescue experiments to establish the functional necessity of AXL. In *Gpr183*^−/−^ T cells, ectopic expression of AXL, but not the empty vector, successfully restored the phosphorylation levels of AXL, JAK2, and STAT5 (Fig. 5J). The restoration of this signaling axis largely rescued T_reg_ differentiation and Foxp3 expression to levels comparable to WT cells (Fig. 5K). Collectively, these data suggest that GPR183-mediated AXL activation plays a necessary role in the JAK2-STAT5 signaling that contributes to the T_reg_ transcriptional program.

### The GPR183-AXL-STAT5 axis mediates HCD-induced T_reg_ accumulation and immune evasion in vivo

Having identified GPR183 as a driver of T_reg_ differentiation, we next sought to determine whether its downstream effector, AXL, is functionally indispensable for GPR183-mediated immune evasion under metabolic stress. To this end, we treated WT and *Gpr183*^Δ*CD4*^ tumor-bearing mice with the specific AXL inhibitor Bemcentinib under either SD or HCD conditions (Fig. 6A). In the MC38 model, HCD significantly accelerated tumor growth in WT mice; however, pharmacological blockade of AXL with Bemcentinib markedly suppressed this HCD-promoted progression, effectively phenocopying the tumor resistance observed in *Gpr183*^Δ*CD4*^ mice (Fig. 6B). Consistent with the tumor growth kinetics, HCD consumption in WT mice triggered a robust accumulation of the intratumoral T_reg_ compartment and up-regulated Foxp3 expression (Fig. 6C). This accumulation was coupled with the hyperactivation of the AXL-JAK2-STAT5 signaling cascade within tumor-infiltrating T_reg_ cells (Fig. 6D). Crucially, administration of Bemcentinib abolished the HCD-induced phosphorylation of AXL, JAK2, and STAT5, subsequently blunting T_reg_ accumulation (WT + HCD versus WT + HCD + Bemcentinib; Fig. 6, C and D). *Gpr183*^Δ*CD4*^ mice maintained consistently low levels of AXL-JAK2-STAT5 signaling regardless of dietary cholesterol intake, remaining largely resistant to HCD-induced T_reg_ accumulation. These findings collectively position AXL as a key downstream mediator of GPR183 in this metabolic-immune cross-talk (Fig. 6, C and D). Last, we evaluated how this GPR183-AXL–mediated T_reg_ response shapes the overall antitumor effector landscape. In HCD-fed WT mice, the intensified T_reg_ presence coincided with impaired CD8^+^ T cell fitness, as reflected by diminished frequencies and reduced production of IFN-γ and GzmB (Fig. 6E). Notably, either pharmacological inhibition of AXL or genetic ablation of GPR183 significantly bolstered the recruitment and cytotoxic capacity of CD8^+^ T cells, effectively counteracting the immunosuppressive state induced by high cholesterol (Fig. 6E). Together, these in vivo data support that the GPR183-AXL-STAT5 axis may represent a pivotal molecular rheostat that links systemic cholesterol metabolism to T_reg_-mediated immune suppression.

**Fig. 6.**
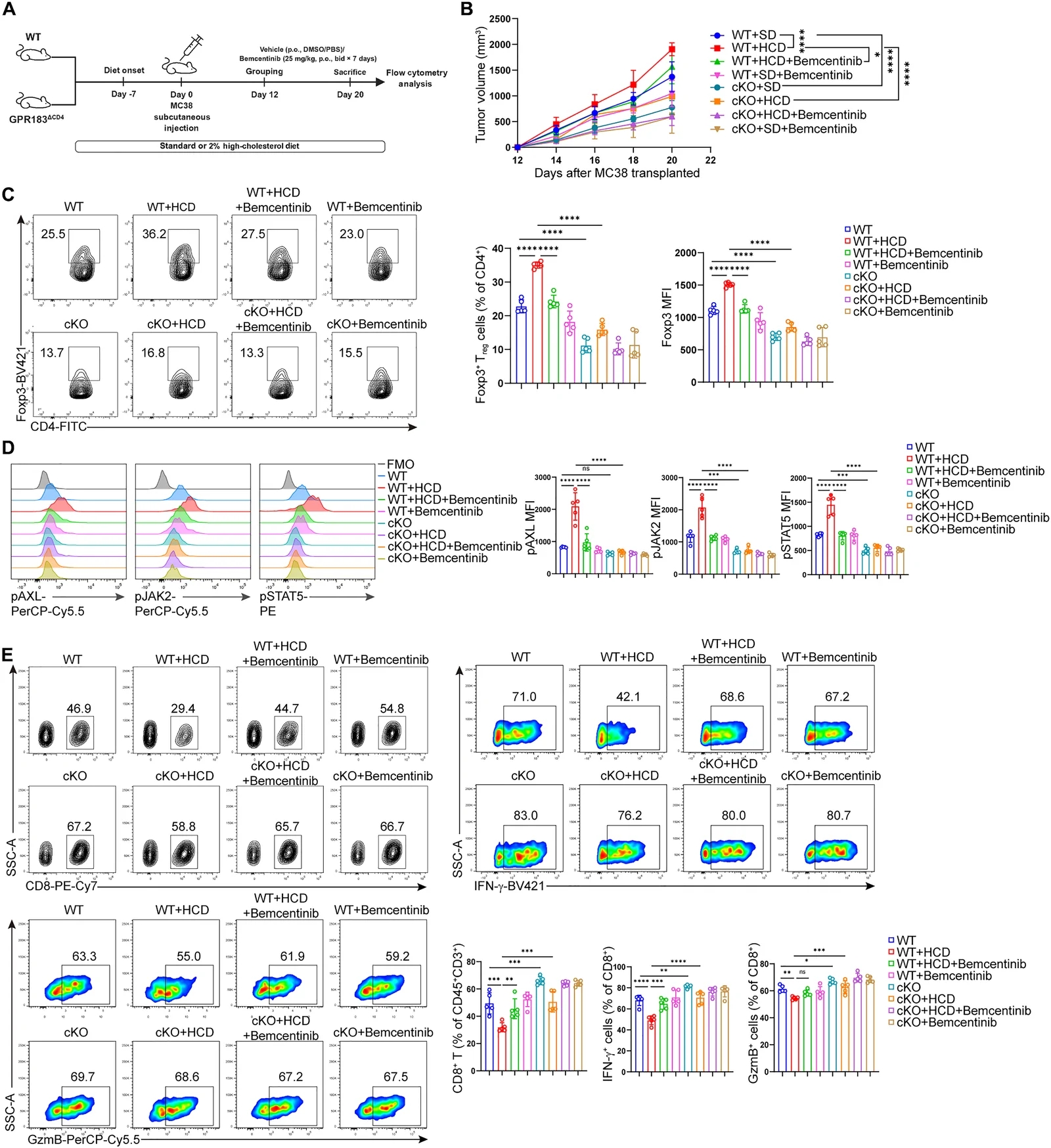
The GPR183-AXL-STAT5 axis mediates HCD-induced T_reg_ accumulation and tumor immune evasion in vivo. (**A**) Schematic illustration of the experimental design. Created in BioRender. A. Qu (2026); https://BioRender.com/0fx7i1b. WT and *Gpr183*^Δ*CD4*^ mice were fed an SD or an HCD and inoculated with MC38 tumor cells, with or without AXL inhibitor (Bemcentinib) treatment. (**B**) Tumor growth curves of MC38 tumors in the indicated groups of mice over 20 days. (**C**) Representative flow cytometry plots and statistical quantification of Foxp3^+^ T_reg_ frequencies and Foxp3 MFI in TILs. (**D**) Phosflow analysis showing the MFI of p-AXL, p-JAK2, and p-STAT5 in tumor-infiltrating CD4^+^ T cells across the experimental groups. (**E**) Representative flow cytometry plots and quantification of intratumoral CD8^+^ T cell frequencies (left) and the proportions of IFN-γ^+^ and GzmB^+^ cells among CD8^+^ T cells (right) in tumors. Data in (B) to (E) are presented as means ± standard deviation (*n* = 5 biological replicates, mice per group). Statistical significance was determined by two-way ANOVA followed by Tukey’s post hoc test. *P < 0.05; **P < 0.01; ***P < 0.001; ****P < 0.0001.

### Pharmacological targeting of GPR183 provides a modest, model-dependent combination benefit with anti-PD-1 therapy

Given the pivotal role of the GPR183-AXL axis in driving T_reg_-mediated immune evasion, we last explored the translational potential of targeting GPR183 to sensitize tumors to immune checkpoint blockade (ICB) under hypercholesterolemic conditions. WT mice were fed an HCD and treated with the GPR183 antagonist NIBR189, anti-PD-1, or a combination of both (Fig. 7A). In the MC38 model, the combination of NIBR189 and anti-PD-1 exhibited superior antitumor efficacy, resulting in the most significant reduction in tumor volume and achieving the highest rate of complete regression compared to either monotherapy (Fig. 7B). At the endpoint (day 22), tumor growth inhibition (TGI) relative to NC was 68.0% for NIBR189, 86.5% for anti-PD-1, and 98.8% for the combination. The combination thus provided an additional 12.3% increase in TGI compared to anti-PD-1 alone (ΔTGI = +12.3%). Bliss independence analysis further supported a modest synergistic interaction in the MC38 model, with the observed combination inhibition (98.8%) exceeding the expected additive effect (95.7%) and yielding a positive Bliss synergy score of +0.0311 (+3.11%). To explore the immunological basis underlying this enhanced therapeutic efficacy, we next analyzed the intratumoral immune landscape by flow cytometry. Flow cytometry analysis revealed that NIBR189 successfully counteracted HCD-induced T_reg_ accumulation (Fig. 7C). Notably, T_reg_ cells in the combination group displayed significantly lower PD-1 expression relative to monotherapy groups, suggesting a marked attenuation of their suppressive identity (Fig. 7D). Concurrently, the combination group attained the highest frequency of tumor-infiltrating CD8^+^ T cells (Fig. 7E). Quantification of the intratumoral CD8^+^ T cell/T_reg_ ratio further demonstrated that all treatment regimens shifted the immune balance toward a more favorable antitumor state, with significantly higher ratios than those observed in the NC group (Fig. 7F). Despite comparable CD8^+^ T cell/T_reg_ ratios among the treatment groups, the combination therapy induced the strongest functional activation of CD8^+^ T cells, as evidenced by increased IFN-γ expression (Fig. 7, G and H). These results demonstrate that in the immunologically “hot” MC38 model, GPR183 inhibition enhances ICB efficacy by simultaneously alleviating T_reg_-mediated suppression and amplifying the effector potency of CD8^+^ T cells.

**Fig. 7.**
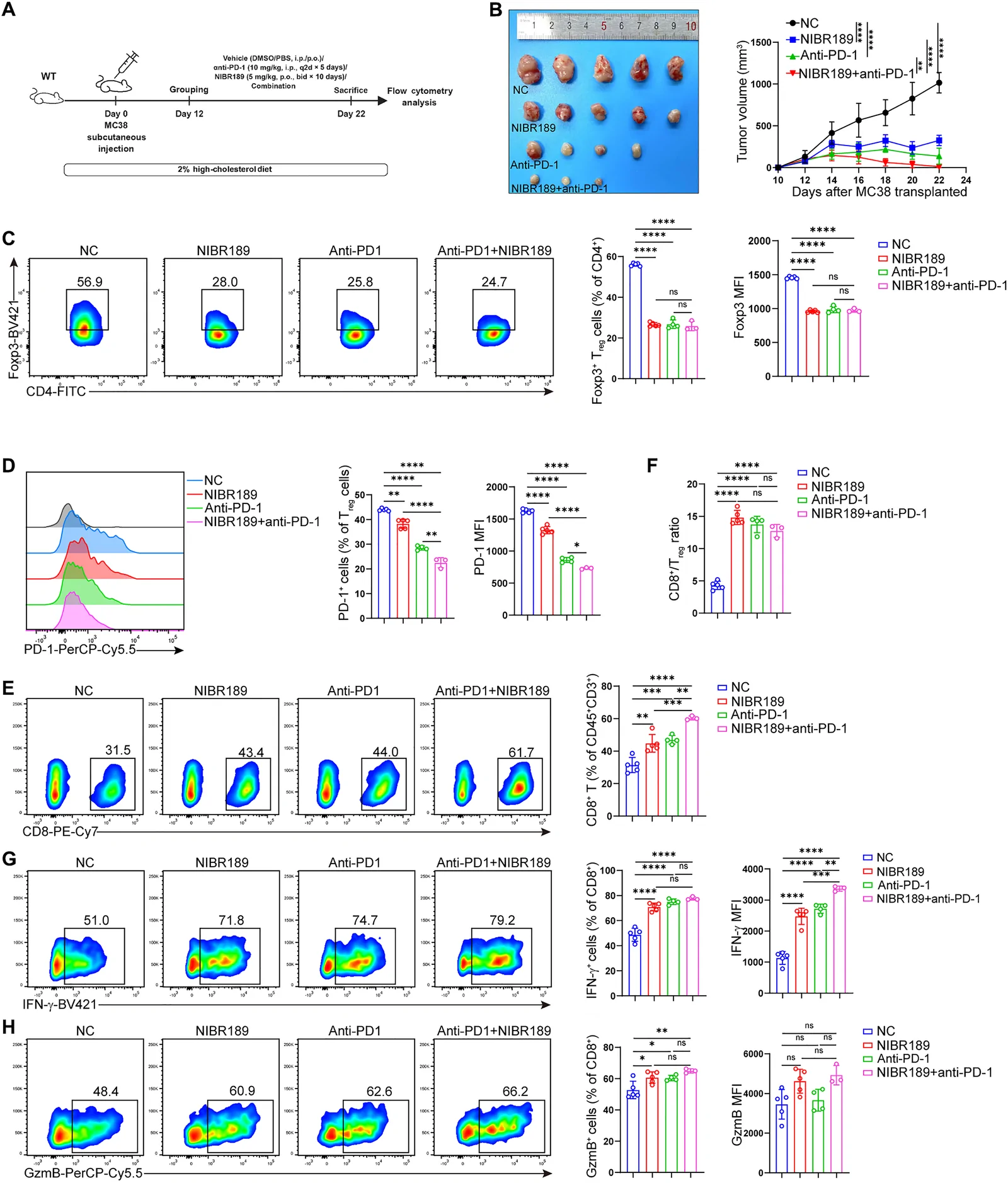
GPR183 blockade shows modest synergy with anti-PD-1 therapy in the MC38 model. (**A**) Schematic illustration of the experimental design. Created in BioRender. A. Qu (2026); https://BioRender.com/rikjzov. WT mice were fed an HCD, transplanted with MC38 cells, and subsequently treated with vehicle (NC, negative control), the GPR183 antagonist NIBR189, anti-PD-1, or a combination of NIBR189 and anti-PD-1. (**B**) Representative images of harvested MC38 tumors (left) and tumor growth curves (right) in the indicated groups. (**C** and **D**) Representative flow cytometry plots (left) and quantification (right) of intratumoral T_reg_ frequencies and Foxp3 MFI (C), alongside PD-1 expression (frequency and MFI) on T_reg_ cells (D). (**E**) Representative flow cytometry plots and quantification of tumor-infiltrating CD8^+^ T cell frequencies. (**F**) Intratumoral CD8^+^ T cell/T_reg_ ratio in the indicated treatment groups. (**G** and **H**) Representative flow cytometry plots and quantification (frequency and MFI) of IFN-γ (G) and GzmB (H) expression in intratumoral CD8^+^ T cells. Data in (B) to (H) are presented as means ± standard deviation (*n* = 5 biological replicates, mice per group). Statistical significance was determined by two-way ANOVA followed by Tukey’s post hoc test. *P < 0.05; **P < 0.01; ***P < 0.001; ****P < 0.0001; ns, not significant.

To further validate the therapeutic potential of GPR183 blockade, we used the B16F10 melanoma model, characterized by its inherently lower immunogenicity and distinct metabolic profile. In this setting, NIBR189 monotherapy led to a modest reduction in tumor growth, while both anti-PD-1 and combination therapies exhibited significant and comparable inhibitory effects relative to the NC group (fig. S8A). Notably, flow cytometry analysis revealed that anti-PD-1 monotherapy triggered a robust compensatory surge in tumor-infiltrating T_reg_ cells—a common mechanism of therapeutic resistance (*31*–*33*)—which was effectively abrogated by NIBR189 cotreatment (fig. S8B). This reduction in T_reg_ frequency was accompanied by a decrease in Foxp3 MFI; while PD-1 levels in the combination group remained comparable to those in the anti-PD-1 monotherapy group, the overall contraction of the T_reg_ pool suggests a significant attenuation of the suppressive burden within the TME (fig. S8, B and C). Although the combination group did not further amplify the CD8^+^ T cell effector response (IFN-γ^+^ and GzmB^+^) beyond the levels achieved by anti-PD-1 alone, it successfully maintained a favorable CD8^+^/T_reg_ ratio (fig. S8, D to G). These data suggest that while GPR183 inhibition effectively prevents therapy-induced T_reg_ expansion, its therapeutic benefit in combination with ICB is context-dependent, likely modulated by the baseline immunogenicity and metabolic characteristics of the tumor model.

## DISCUSSION

Our findings delineate a mechanism by which the cholesterol metabolite 7α,25-OHC, acting through its receptor GPR183, promotes T_reg_-mediated tumor immune evasion. The clinical relevance of this pathway is supported by analyses of human GC specimens. Rather than serving as a passive metabolic byproduct, this oxysterol functions as an instructive signal that directly promotes T_reg_ differentiation and fortifies their suppressive identity, thereby establishing a T_reg_-dominated immunological barrier within the TME. These findings highlight metabolic-immune cross-talk as an important regulatory layer in T_reg_ biology and reveal a potential therapeutic vulnerability.

### A stromal-metabolic niche for T_reg_ instruction

Our clinical data reveal a spatial dichotomy: the machinery for 7α,25-OHC synthesis (CH25H, CYP7B1) is predominantly housed within Vimentin^+^ stromal cells, while the receptor GPR183 is expressed on tumor-infiltrating T_reg_ cells. This compartmentalization suggests a paracrine model wherein stromal cells, responding to a cholesterol-rich milieu, create a local oxysterol gradient that promotes T_reg_ differentiation and intratumoral accumulation. While a minor contribution from tumor cells cannot be excluded, the bulk of 7α,25-OHC synthesis is consistently attributed to stromal cells. Direct LC-MS/MS quantification confirmed the presence of 7α,25-OHC in human GC tissues, providing biochemical evidence that this oxysterol is indeed produced within the TME. The finding that the proportion of GPR183^+^ T_reg_ cells, but not of enzyme-expressing Vimentin^+^ stromal cells alone, is associated with poor prognosis underscores a crucial principle: The presence of a metabolic signal is necessary but insufficient; suppression is dictated by the presence of immune cells capable of sensing that signal. This stromal-immune metabolic coupling provides a mechanistic basis for the long-observed link between hypercholesterolemia and cancer progression.

### GPR183 as a “metabolic rheostat” for T_reg_ lineage commitment

Our data establish GPR183 as a critical rheostat linking systemic and local cholesterol levels to T_reg_ fate. Using a suite of conditional knockout (KO) mice, we demonstrated that GPR183 is required cell-intrinsically for the accumulation of the intratumoral T_reg_ pool under hypercholesterolemic conditions. Crucially, the effect is not limited to de novo differentiation; GPR183 is equally indispensable for maintaining the suppressive identity of mature T_reg_ cells within the TME. Ablation of GPR183 specifically in T_reg_ cells led to a loss of Foxp3 expression, down-regulation of key effector molecules (PD-1 and IL-10), and a consequent unleashing of CD8^+^ T cell cytotoxicity. This indicates that continuous oxysterol sensing is required to sustain the suppressive program and that the metabolic niche actively prevents T_reg_ destabilization. Together with its role in promoting T_reg_ differentiation and Foxp3 expression, GPR183 also supports the maintenance and functional adaptation of intratumoral T_reg_ cells—primarily by sustaining local proliferation rather than by dictating recruitment, retention, or survival—positioning it as a regulator of T_reg_ adaptation within the TME beyond its canonical role in immune trafficking. The consequent restoration of CD8^+^ T cell function upon GPR183 loss, without directly targeting CD8^+^ T cells, highlights a “suppressor-first” hierarchy in this metabolic immune-evasion strategy.

### The GPR183-AXL-JAK2-STAT5 signaling axis: A molecular bridge from lipid sensing to transcriptional programming

Mechanistically, we identified a signaling cascade linking GPR183 to the core T_reg_ transcriptional machinery. Upon 7α,25-OHC stimulation, GPR183 engagement leads to the up-regulation and rapid activation of the RTK AXL. The swift kinetics of AXL, JAK2, and STAT5 phosphorylation suggest a preassembled or highly poised signaling module. Our findings demonstrate that GPR183 recruits SFKs as a biochemical bridge to transactivate AXL, a mechanism that explains the near-instantaneous onset of the downstream cascade upon ligand binding. Our genetic and pharmacological data support that AXL is not a passive bystander but an important signal transducer. The observation that AXL expression itself is induced by GPR183 activation may create a positive feedback loop, sensitizing T cells to subsequent oxysterol signals and reinforcing lineage stability. The successful rescue of the T_reg_ differentiation defect in *Gpr183*^−/−^ T cells by ectopic AXL expression suggests its position as a key mediator downstream of the receptor. This pathway—linking a GPCR to an RTK to drive a specific lineage program—represents a departure from classical models of cytokine-driven T cell differentiation and identifies a direct molecular interface between lipid metabolism and immune cell fate.

### Translational implications: Targeting the metabolic checkpoint to enhance immunotherapy

Our findings have direct therapeutic implications. In the context of hypercholesterolemia, which frequently accompanies Western diets and may also arise during cancer progression or treatment, anti-PD-1 monotherapy may be insufficient to overcome immune suppression (*34*–*36*). While PD-1 blockade can reinvigorate exhausted effector T cells, it does not address the continuous metabolic replenishment of the suppressive T_reg_ compartment. Consistent with this concept, anti-PD-1 treatment induced a compensatory expansion of intratumoral T_reg_ cells in the B16F10 model, a recognized mechanism of adaptive resistance to ICB (*31*–*33*). Pharmacological inhibition of GPR183 with NIBR189 effectively prevented this therapy-induced T_reg_ expansion, thereby disrupting an upstream metabolic pathway that sustains immune suppression.

An important observation from our study is that the combination benefit of GPR183 blockade with anti-PD-1 therapy was modest and model-dependent. NIBR189 enhanced the efficacy of anti-PD-1 therapy in the immunologically “hot” MC38 model, whereas its additional benefit in the poorly immunogenic B16F10 model was limited. This difference likely reflects the distinct baseline immune landscapes of these tumors. MC38 tumors contain abundant preexisting T cell infiltration, allowing the reduction of T_reg_-mediated suppression to translate directly into enhanced CD8^+^ T cell function and superior tumor control. In contrast, although GPR183 inhibition effectively reduced the suppressive T_reg_ burden in B16F10 tumors, the intrinsically limited T cell infiltration and lower immunogenicity of this model may constrain the conversion of immune relief into additional antitumor activity. These findings suggest that targeting the 7α,25-OHC–GPR183 axis may be particularly beneficial in tumors characterized by active immune engagement but substantial T_reg_-mediated suppression. More broadly, our results identify metabolic oxysterol signaling as an underappreciated determinant of checkpoint responsiveness and support GPR183 antagonism as a rational strategy to recalibrate the tumor immune microenvironment. Future studies should investigate whether combining GPR183 blockade with approaches that enhance immune priming or T cell recruitment can further improve outcomes in immunologically “cold” tumors.

### Study limitations and future directions

Despite these findings, several limitations should be considered. Although our data identify the 7α,25-OHC–GPR183 axis as an important regulator of T_reg_ differentiation and stability, the precise sources and temporal dynamics of oxysterol production within the human gastric TME remain to be fully defined. In addition, while our mechanistic studies delineate a GPR183-AXL-JAK2-STAT5 signaling pathway in murine models and in vitro systems, further validation in larger patient cohorts and functional analyses using human primary T cells will be necessary to establish its relevance in human disease. Notably, the mechanistic and therapeutic investigations in this study were primarily performed in MC38 and B16F10 models rather than dedicated GC models. Although these syngeneic systems enabled us to interrogate the immunoregulatory role of the 7α,25-OHC–GPR183 axis across distinct immune contexts, future validation in GC-specific models will be important to confirm the generalizability of our findings to gastric tumor biology. Moreover, the therapeutic potential of targeting GPR183 requires evaluation in preclinical models that more closely recapitulate the metabolic heterogeneity of human tumors. Addressing these questions will be important for translating metabolic checkpoint targeting into clinical practice. Future studies testing pharmacological GPR183 inhibitors in combination with ICB in clinically relevant models will help determine the feasibility of this therapeutic strategy.

## MATERIALS AND METHODS

### Experimental design

This study was designed to investigate the role of the 7α,25-OHC/GPR183 axis in modulating T_reg_-mediated immune evasion in GC. We used a combination of human clinical specimen analysis, conditional KO mouse models (CD4-specific and T_reg_-specific *Gpr183* deficiency), and in vitro mechanistic assays. The primary objectives were to identify the cellular sources of oxysterol ligands, demonstrate how the 7α,25-OHC/GPR183 axis regulates T_reg_ differentiation and immunosuppressive function, characterize the GPR183-AXL signaling cascade in T_reg_ cells, and evaluate the therapeutic potential of GPR183 inhibition in sensitizing tumors to anti-PD-1 therapy. Mice were randomized into treatment groups, and researchers were blinded during tumor measurement where feasible.

### Human samples and mIF

Peripheral blood from three healthy donors was collected for in vitro T_reg_ differentiation. FFPE tumor tissues from 93 patients with GC, all of whom underwent curative (R0) resection and had not received neoadjuvant therapy, were used for mIF analyses. OS was defined from surgery to death or last follow-up. mIF (Opal 6-Plex, Akoya) detected CD4, Foxp3, GPR183, Vimentin, CH25H, and CYP7B1. Detailed information for all antibodies and kits used is provided in table S3. Imaging was performed on TissueFAXS SPECTRA and analyzed with StrataQuest; patients were stratified by median expression.

### Mice

CD45.1 mice (catalog no. NM-KI-210226) and *Gpr183*-KO mice (catalog no. NM-KO-190285) were obtained from Shanghai Model Organisms Center, Inc. *Gpr183*^fl/fl^ mice (strain no. S-CKO-10527) and *CD4*^Cre^*Gpr183*^fl/fl^ mice (stock no. C001287) were purchased from Cyagen. To generate T_reg_-specific *Gpr183*-deficient mice, *Foxp3*^Cre^ mice (the Jackson Laboratory, strain no. 016959) were crossed with *Gpr183*^fl/fl^ mice to yield *Foxp3*^Cre^*Gpr183*^fl/fl^ offspring. All experimental mice used in this study were male.

### Genotyping of conditional KO mice

Tail DNA was extracted using the TIANamp Genomic DNA Kit (TIANGEN), and genotyping polymerase chain reaction was performed using TaKaRa Taq V.2.0. Primer sequences are listed in table S4.

### Cell lines

MC38 cells were obtained from the National Biomedical Cell Resource Center (BMCR), National Infrastructure of Cell Line Resource (NICR; Beijing, China; catalog no. 1101MOU-PUMC000523), and B16-F10 cells were obtained from American Type Culture Collection (ATCC; catalog no. CRL-6475). MC38 and B16-F10 cells were cultured in RPMI-1640 and Dulbecco’s modified Eagle’s medium, respectively, supplemented with 10% fetal bovine serum and 1% penicillin-streptomycin in a humidified atmosphere containing 5% CO_2_ at 37°C. Cells were detached using trypsin-EDTA and passaged at ∼70 to 80% confluence according to standard cell culture procedures. All cell lines were routinely tested for mycoplasma contamination and confirmed to be negative before experimental use.

### In vitro T_reg_ differentiation

Naïve CD4^+^ T cells were isolated from human PBMCs or murine spleens by magnetic bead sorting. Cells were cultured in complete RPMI-1640 medium supplemented with IL-2 (20 IU/ml), transforming growth factor–β (1 ng/ml), β-mercaptoethanol (1:1000), and anti-CD3/CD28 antibodies (1 μg/ml). Treatments included 7α,25-OHC (100 nM), NIBR189 (250 nM), or Bemcentinib (500 nM), according to the experimental design. Cultures were maintained for 72 hours. Detailed information regarding the cell isolation kits, antibodies, small-molecule inhibitors, and cytokines used in this study is provided in tables S5 to S7.

For retroviral AXL overexpression, activated murine naïve CD4^+^ T cells were transduced with pMSCV-Hyg-oeAXL retrovirus using RetroNectin-coated plates, and expression was validated by flow cytometry 48 hours posttransduction.

### Diets and tumor models

Mice were fed an SD or a 2% HCD for 1 week prior to tumor inoculation. Tumors were established by subcutaneous injection of MC38 (5 × 10^5^) or B16-F10 (2 × 10^5^) cells in 100 μl phosphate-buffered saline (PBS). Tumor growth was measured every other day with calipers, and volume was calculated as (*length* × *width*^2^)/2.

For immunotherapy, C57BL/6 mice were randomly assigned to four groups (*n* = 5 per group): vehicle control, NIBR189 alone, anti-PD-1 antibody alone, or the combination (NIBR189 + anti-PD-1). Detailed information regarding the inhibitors and antibodies used in vivo is provided in tables S7 and S8. Anti-PD-1 was administered intraperitoneally at 10 mg/kg every other day for five doses, starting when MC38 tumors reached 80 to 100 mm^3^ or on day 6 post–B16-F10 inoculation. NIBR189 was administered by oral gavage at 5 mg/kg twice daily (bid) for 10 consecutive days. For AXL inhibition, Bemcentinib was administered orally at 25 mg/kg bid.

### Flow cytometry and phospho-flow

Single-cell suspensions from cultured cells or tumor tissues were stained for surface markers, intracellular cytokines, and nuclear transcription factors (including Foxp3) following standard procedures. For intracellular cytokine analysis, cells were stimulated with Cell Activation Cocktail for 6 hours before fixation and permeabilization. Phospho-flow analysis of p-AXL, p-JAK2, and p-STAT5 was performed on naïve CD4^+^ T cells and tumor-infiltrating CD4^+^ T cells. Single-cell suspensions were stained following the manufacturer’s instructions (Invitrogen). Detailed information regarding the flow cytometry antibodies and related kits used is provided in tables S5 and S9. Data were acquired on a BD FACSCanto II and analyzed with FlowJo v10.10.

### Transcriptomic sequencing and analysis

Naïve CD4^+^ T cells from WT and *Gpr183*^−/−^ mice were cultured under T_reg_-polarizing conditions with 7α,25-OHC for 72 hours (*n* = 5 per group). RNA-seq was performed, and DESeq2 identified DEGs [adjusted *P* value < 0.05, log_2_(fold change) ≥ 1.5]. Gene ontology and KEGG analyses were applied to interpret biological functions.

### LC-MS/MS quantification of 7α,25-OHC

Frozen GC tissues from five treatment-naïve patients and independent tumor samples from MC38 tumor-bearing mice maintained on SD or HCD (*n* = 3 per group) were subjected to 7α,25-OHC quantification. Briefly, ∼20 mg of frozen tissue was extracted with ethyl acetate. After centrifugation and evaporation, the dried extracts were reconstituted in methanol and analyzed using a QTRAP 6500+ LC-MS/MS system (AB Sciex) equipped with a Phenomenex Kinetex C18 column. Chromatographic separation was performed using a mobile phase consisting of acetonitrile/water/isopropanol containing 0.04% acetic acid. Mass spectrometry detection was conducted in positive electrospray ionization (ESI) mode using optimized multiple reaction monitoring (MRM) transitions for 7α,25-OHC.

### Statistical analysis

Data were analyzed using GraphPad Prism (v10.6) and are presented as means ± standard deviation. For comparisons between two groups, two-tailed unpaired Student’s *t* test or Mann-Whitney *U* test was used as appropriate. For multiple group comparisons, one-way or two-way analysis of variance (ANOVA) followed by Tukey’s post hoc test was performed. Survival curves were estimated using the Kaplan-Meier method and compared via the log-rank (Mantel-Cox) test. Sample sizes (*n*) and the specific statistical tests used for each experiment are indicated in the corresponding figure legends. *P* < 0.05 was considered statistically significant (*P < 0.05; **P < 0.01; ***P < 0.001; ****P < 0.0001).

### Ethics statement

This study was approved by the Ethics Committee of Tianjin Medical University Cancer Institute and Hospital (approval no. EK20240257). Informed consent was obtained from all participants prior to the collection of human tissue samples. The study was conducted in accordance with institutional guidelines and the principles of the Declaration of Helsinki.

All animal experiments were conducted in accordance with institutional guidelines for the care and use of laboratory animals and were approved by the Institutional Animal Care and Use Committee (IACUC) of Tianjin Medical University Cancer Institute and Hospital (approval no. NSFC-AE-2024306). Mice were maintained under specific pathogen-free conditions throughout the study.

### AI disclosure

The authors used DeepSeek-V3.2 as a writing aid solely for improving the linguistic clarity and correcting grammatical errors. The original scientific content was authored by the researchers, and all AI-suggested refinements were manually reviewed and validated by the authors to ensure accuracy and scientific integrity.
