# Supplementary material for: GPR183 senses 7α,25-OHC to drive Treg differentiation and tumor immune evasion via AXL-JAK2-STAT5 signaling
Source: Sci Adv. 2026 Oct 30;12(44):eaeh9736. doi: 10.1126/sciadv.aeh9736 (PMC13721965; doi:10.1126/sciadv.aeh9736)
Supplement: Supplementary file 1 [file sciadv.aeh9736_sm.pdf]

Science Advances
AAAS

Supplementary Materials for

GPR183 senses 7α,25-OHC to drive T_{reg} differentiation and tumor immune evasion via AXL-JAK2-STAT5 signaling

Yang Qu *et al.*

Corresponding author: Hui Li, lihui05@tmu.edu.cn

Sci. Adv. **12**, eaeh9736 (2026)
DOI: 10.1126/sciadv.aeh9736

This PDF file includes:

Figs. S1 to S8
Tables S1 to S9

Supplementary Materials

Figure S1.

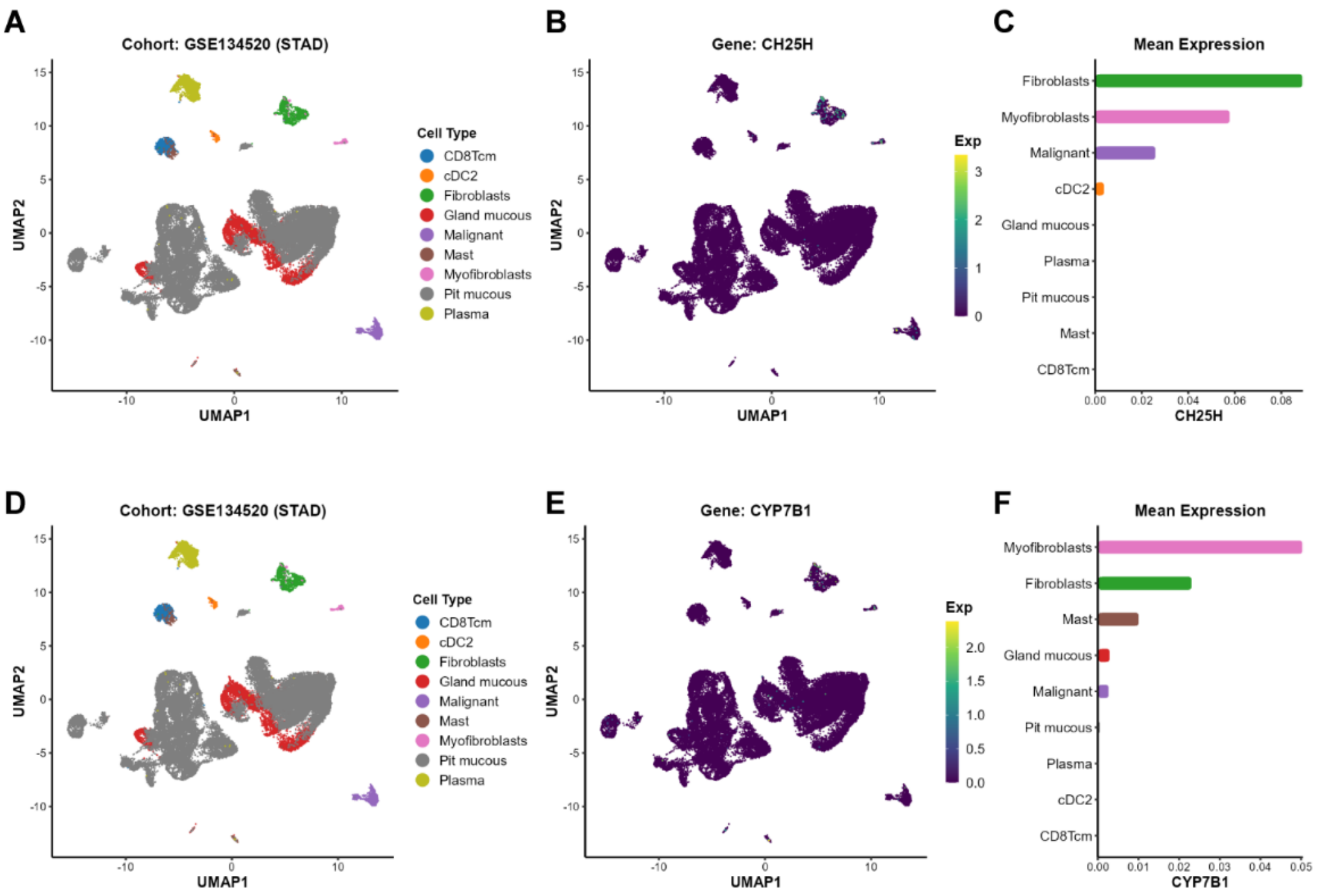

G

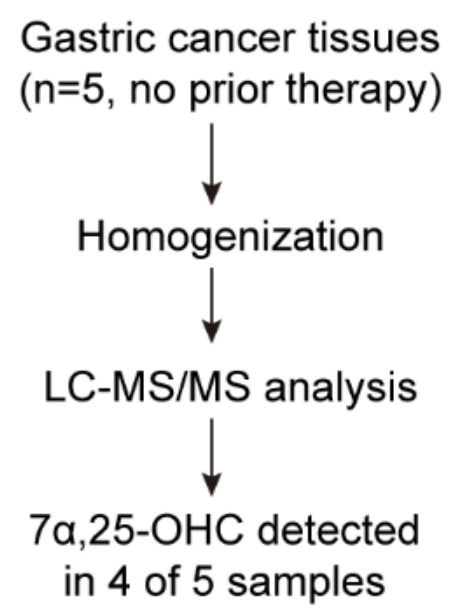

Sample ID	7α,25-OHC(ng/g)
T1	1.74
T2	1.85
T3	0.00
T4	0.68
T5	1.03

Figure S1. Single-cell transcriptomic identification of fibroblasts as the source of CH25H/CYP7B1 and direct LC-MS/MS quantification of 7α,25-OHC in human gastric cancer tissues. Single-cell RNA sequencing data from the GSE134520 (STAD) dataset were analyzed via the Tumor Immune Single-cell Hub (TISCH) database. (A, D) UMAP plots showing the clustering and annotation of major cell types in the gastric adenocarcinoma microenvironment. (B, E) Feature plots showing the expression patterns and distribution of CH25H (B) and CYP7B1 (E) across different cell clusters. (C, F) Bar plots showing the mean expression levels of CH25H (C) and CYP7B1 (F) in each cell type, with preferential enrichment in fibroblasts and myofibroblasts. (G) LC-MS/MS quantification of 7α,25-OHC in

freshly frozen gastric cancer tissues from five treatment-naïve patients. 7α,25-OHC was detected in four of five samples, with concentrations ranging from 0.68 to 1.85 ng/g tissue; the concentration in sample T3 was 0 ng/g tissue.

Figure S2.

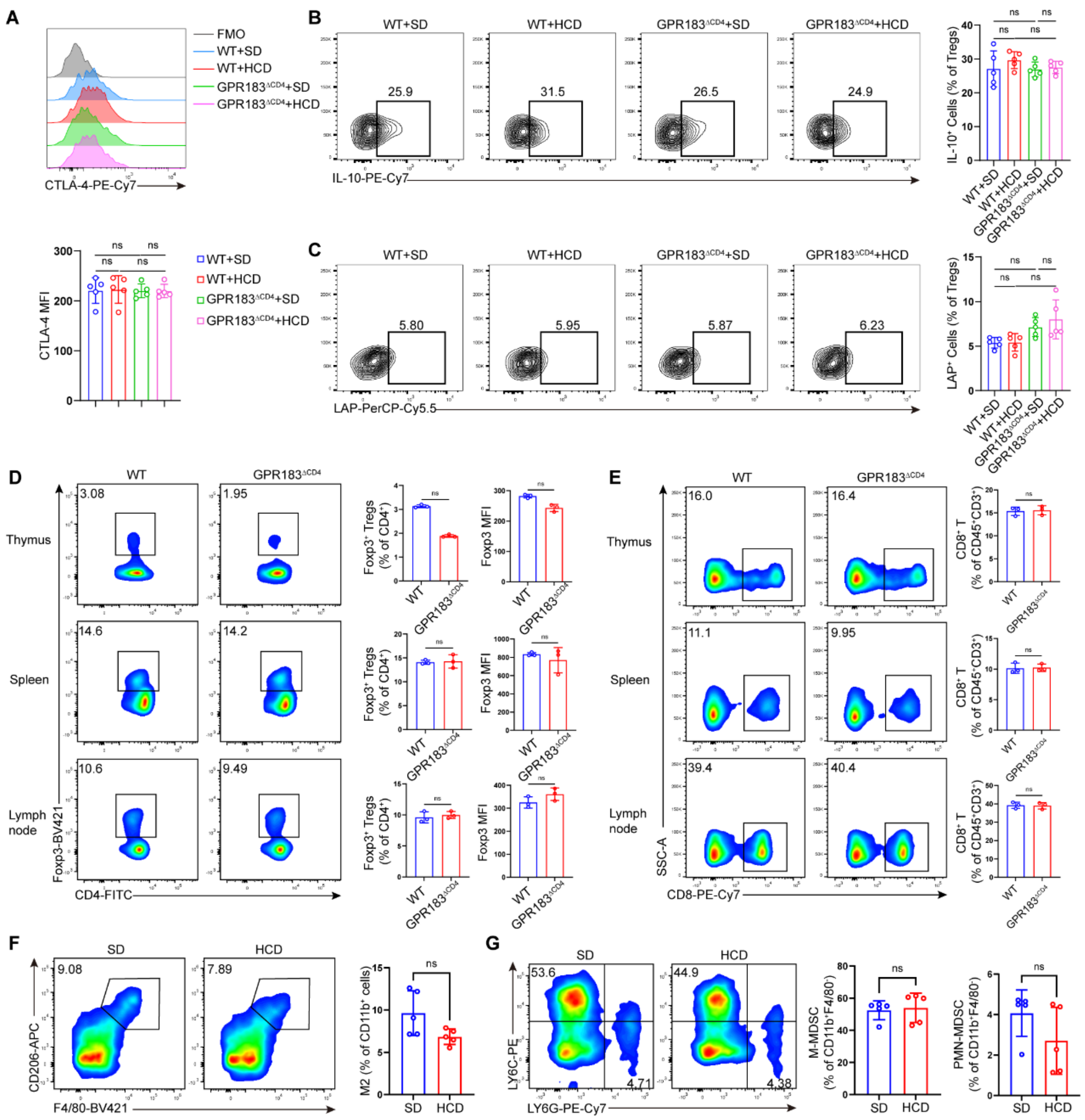

Figure S2. Immune profiling in Gpr183$^{\triangle CD4}$ mice and effect of HCD on myeloid subsets. (A-C) Functional phenotypic analysis of tumor-infiltrating Tregs. Representative flow cytometry plots and quantification of (A) CTLA-4 MFI, (B) the frequency of IL-10^{+} cells, and (C) the frequency of LAP^{+} cells among Tregs isolated from the TME of WT and Gpr183$^{\triangle CD4}$ mice fed with either SD or HCD. Data are presented as mean ± SD (n = 5 biological

replicates, mice per group). Statistical significance was determined by two-way ANOVA followed by Tukey's post hoc test. (D) Representative flow cytometry plots (left) and quantification (right) of Treg frequencies and Foxp3 MFI in the thymus, spleen, and lymph nodes of WT and Gpr183$^{\triangle CD4}$ mice. (E) Representative flow cytometry plots (left) and frequencies (right) of $CD8^+$ T cells in the thymus, spleen, and lymph nodes of WT and Gpr183$^{\triangle CD4}$ mice. Data in (D and E) are presented as mean ± SD (n = 3 biological replicates, mice per group). Statistical significance was determined by unpaired Student's t test. (F) Flow cytometric analysis of M2-type macrophages (gated as $CD11b^+F4/80^+CD206^+$) among $CD11b^+$ cells in MC38 tumors from SD- or HCD-fed WT mice. (G) Analysis of monocytic MDSCs (M-MDSC; $CD11b^+Ly6G^-Ly6C^+$) and polymorphonuclear MDSCs (PMN-MDSC; $CD11b^+Ly6G^+Ly6C^-$). Data in (F and G) are presented as mean ± SD (n = 5 biological replicates, mice per group). Statistical significance was determined by unpaired Student's t test. *P < 0.05, **P < 0.01, ***P < 0.001, ****P < 0.0001; ns, not significant.

Figure S3.

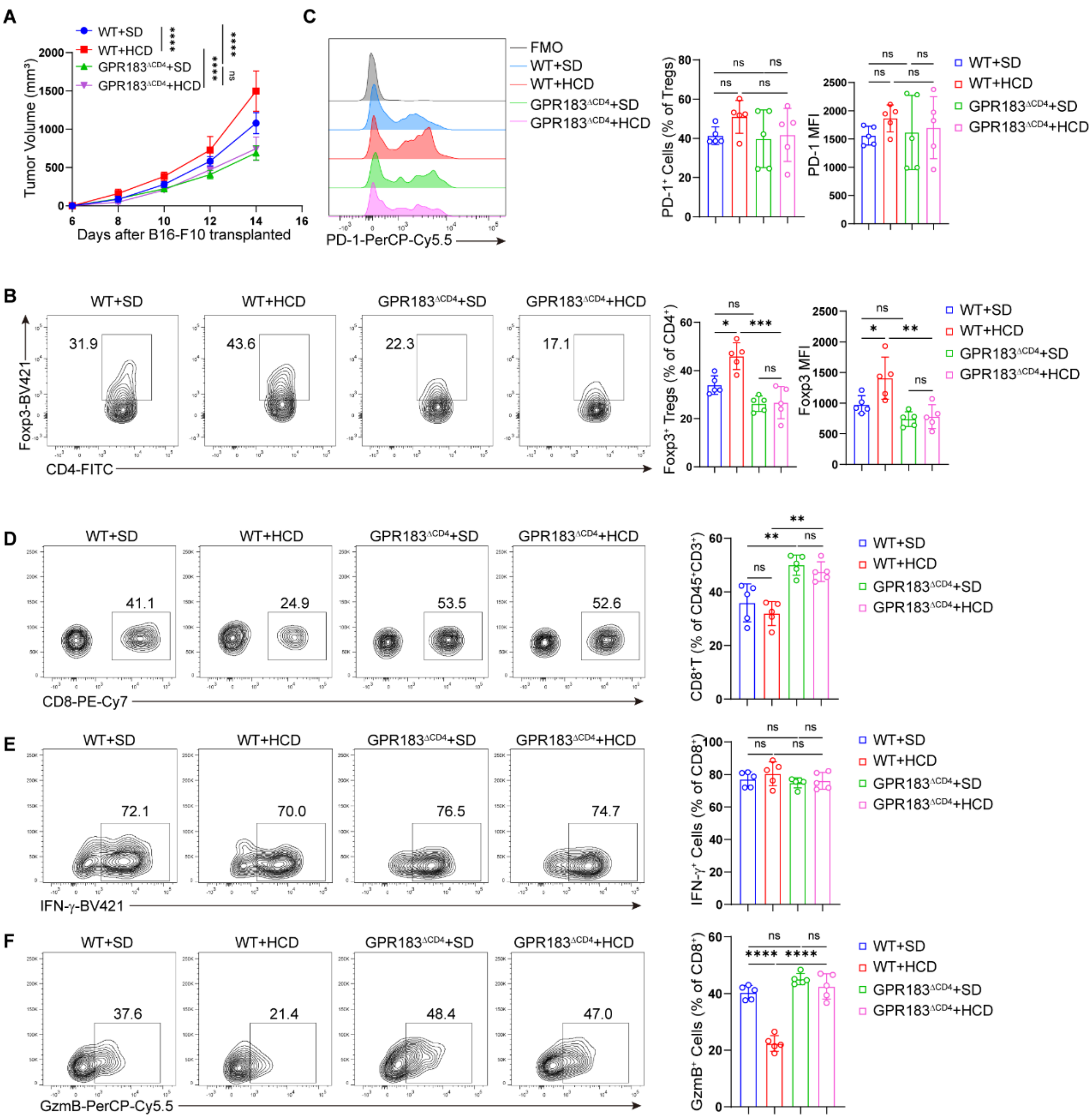

Figure S3. GPR183 in CD4⁺ T cells is required for cholesterol-induced tumor growth and immunosuppression in the B16F10 melanoma model. (A) B16F10 tumor growth curves in WT and Gpr183$^{\triangle CD4}$ mice fed with a standard diet (SD) or a high-cholesterol diet (HCD). (B) Representative flow cytometry plots and quantification of intratumoral Treg frequencies and Foxp3 MFI in the indicated groups. (C) Representative flow cytometry histograms and quantification (frequency and MFI) of PD-1 expression on intratumoral Tregs. (D) Representative flow cytometry plots and frequencies of intratumoral CD8⁺ T cells. (E, F) Representative flow cytometry plots and quantification (frequency and MFI) of effector

molecules IFN-γ (E) and GzmB (F) in intratumoral $CD8^+$ T cells. Data in (A–F) are presented as mean ± SD (n = 5 biological replicates, mice per group). Statistical significance was determined by two-way ANOVA followed by Tukey's post hoc test. *$P < 0.05$, **$P < 0.01$, ***$P < 0.001$, ****$P < 0.0001$; ns, not significant.

Figure S4.

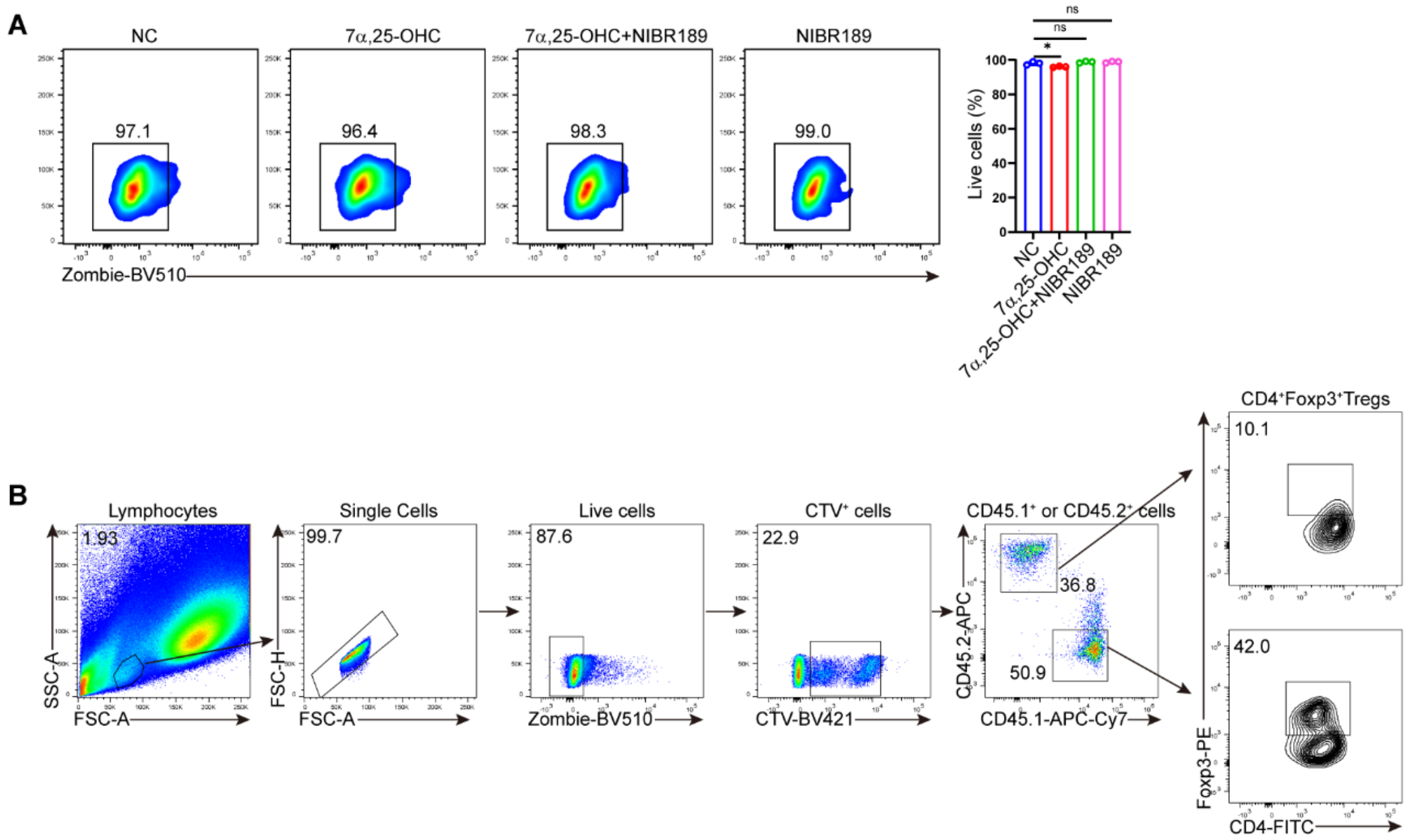

Figure S4. Validation of the GPR183-7α,25-OHC axis components and experimental gating strategies. (A) Representative flow cytometry plots (left) and quantification (right) of cell viability. Naïve $CD4^+$ T cells were treated with 7α,25-OHC (100 nM) in the presence or absence of the GPR183 antagonist NIBR189 (250 nM) for 72 hours. Viability was determined by Zombie-BV510 negative staining. Data are presented as mean ± SD (n = 3 biological replicates). (B) Comprehensive flow cytometry gating strategy for the competitive adoptive transfer assay. Donor cells were sequentially identified by gating on lymphocytes, single cells, and live cells. The adoptively transferred population was defined by CellTrace Violet (BV421) positivity. Within this gate, WT ($CD45.1^+$) and $Gpr183^{-/-}$ ($CD45.2^+$) $CD4^+$ TILs were distinguished to analyze their respective $Foxp3^+$ Treg differentiation frequencies within the MC38 tumor microenvironment.

Figure S5.

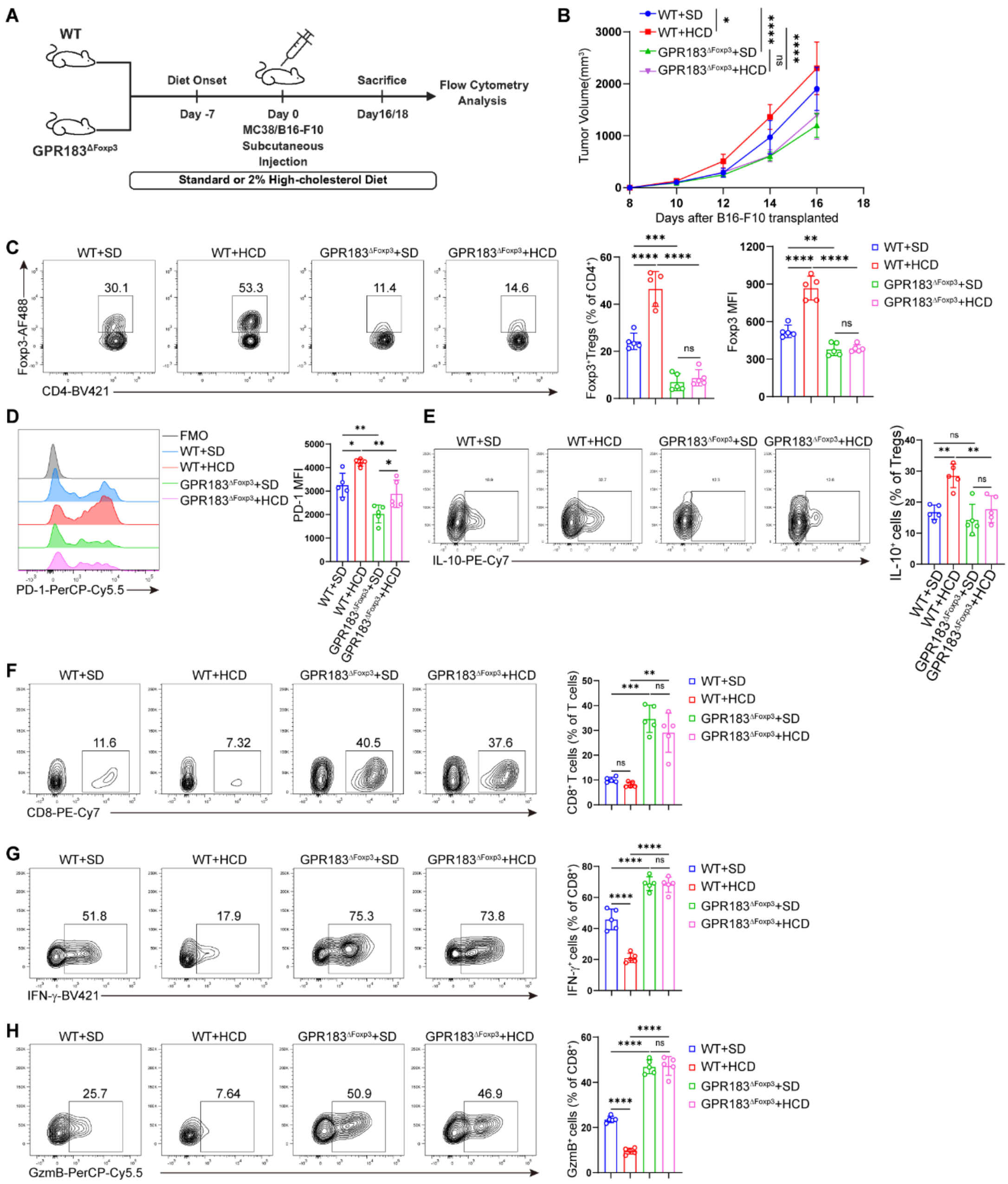

Figure S5. GPR183 is required for the suppressive identity and functional stability of mature Tregs in the B16F10 melanoma model. (A) Schematic illustration of the experimental design. Created in BioRender. Qu, A. (2026) https://BioRender.com/6qf7ut5. WT and Gpr183$^{\triangle Foxp3}$ mice were fed a standard diet (SD) or a high-cholesterol diet (HCD) starting 7 days prior to subcutaneous B16F10 tumor cell injection. (B) B16F10 tumor growth curves in the indicated groups. (C) Representative flow cytometry plots (left) and

quantification (right) of intratumoral Treg frequencies and Foxp3 MFI. (D, E) Representative flow cytometry histograms or plots and quantification (frequency or MFI) of suppressive markers PD-1 (D) and IL-10 (E) in intratumoral Tregs. (F) Representative flow cytometry plots and frequencies of intratumoral $CD8^+$ T cells. (G, H) Representative flow cytometry plots and quantification (frequency) of effector molecules IFN-γ (G) and GzmB (H) in intratumoral $CD8^+$ T cells. Data in (B–H) are presented as mean ± SD (n = 5 biological replicates, mice per group). Statistical significance was determined by two-way ANOVA followed by Tukey's post hoc test. *$P < 0.05$, **$P < 0.01$, ***$P < 0.001$, ****$P < 0.0001$; ns, not significant.

Figure S6.

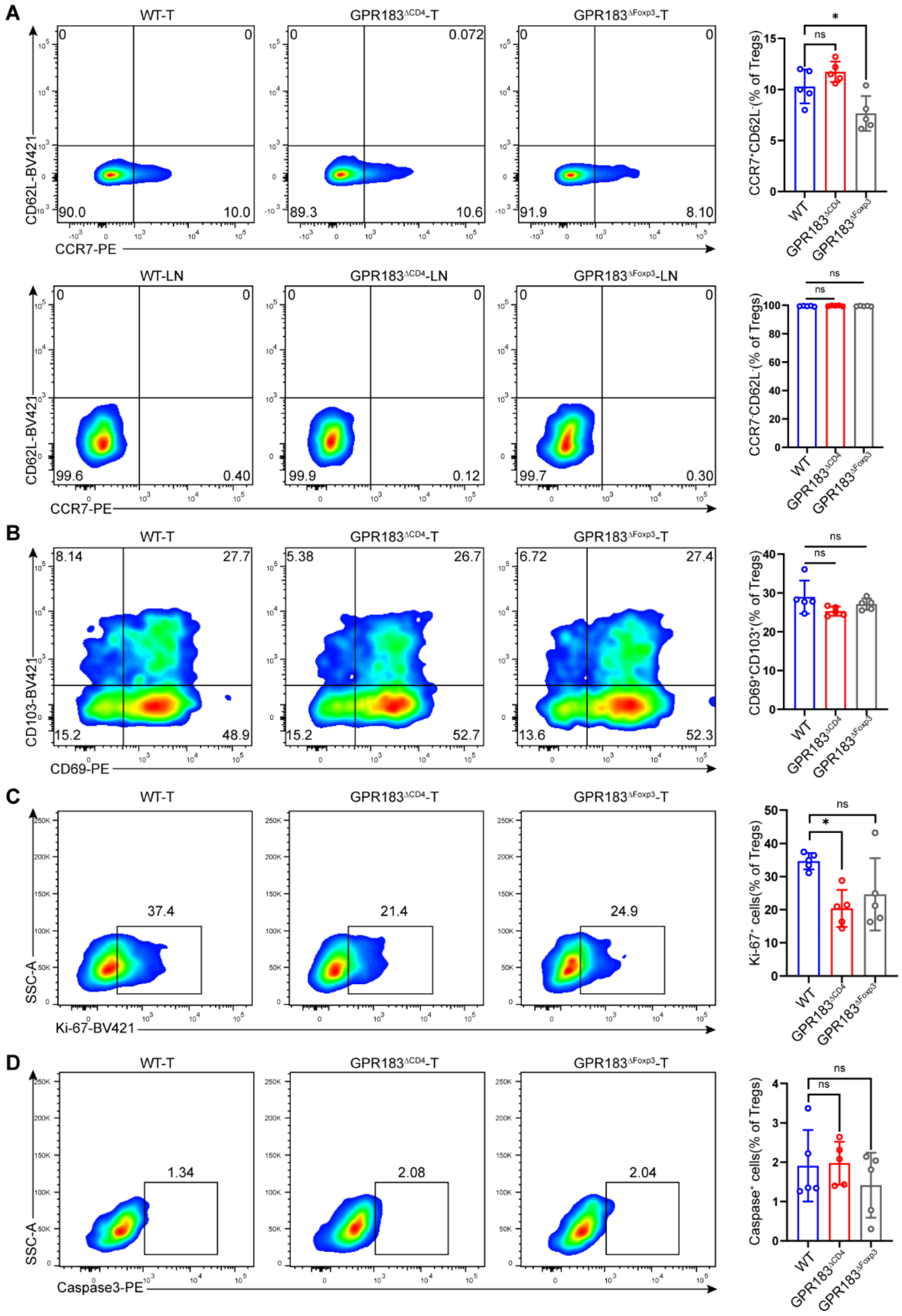

Figure S6. GPR183 supports intratumoral Treg accumulation independently of altered trafficking, tissue residency, or apoptosis. (A) Representative flow cytometry plots and

quantification of CCR7 and CD62L expression on CD4$^+$Foxp3$^+$ Tregs from MC38 tumors (upper) and tumor-draining lymph nodes (lower). (B-D) Representative flow cytometry plots and quantification of CD69/CD103 (B), Ki-67 (C), and cleaved caspase-3 (D) expression on tumor-infiltrating CD4$^+$Foxp3$^+$ Tregs. Data were obtained from WT, Gpr183$^{\Delta CD4}$, and Gpr183$^{\Delta Foxp3}$ mice. Data are presented as mean ± SD (n = 5 biological replicates, mice per group). Statistical significance was determined by one-way ANOVA followed by Tukey's post hoc test. *$P < 0.05$; ns, not significant.

Figure S7.

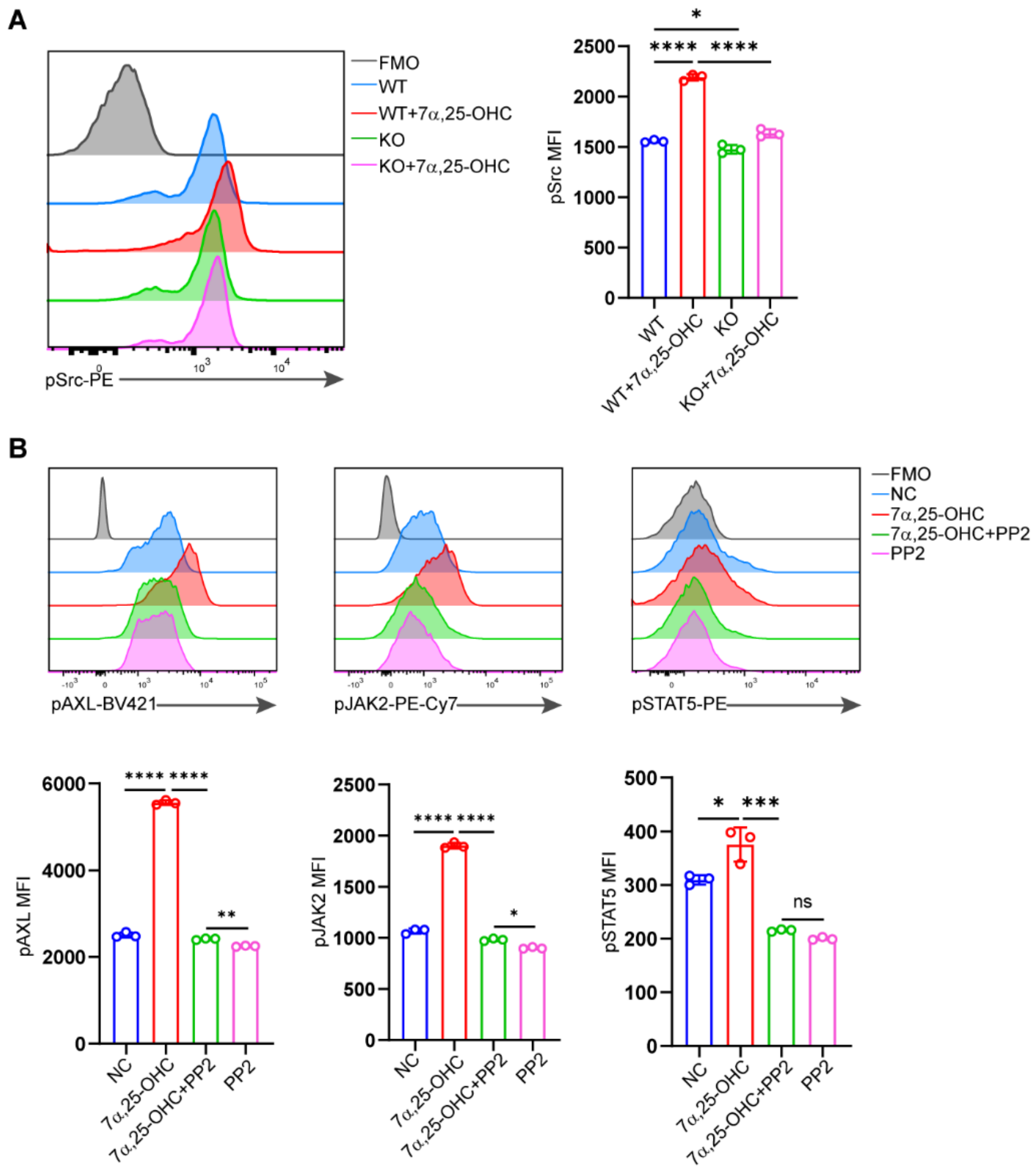

Figure S7. GPR183 activates the AXL-JAK2-STAT5 axis via Src family kinases.

(A) Representative histograms and quantification of phospho-Src (pSrc) (MFI) in WT and Gpr183$^{-/-}$ naïve CD4$^+$ T cells treated with 7α,25-OHC for 5 min. Data are presented as mean ±

SD (n = 3 biological replicates). (B) Representative histograms and quantification of pAXL, pJAK2, and pSTAT5 (MFI) in WT naïve CD4$^+$ T cells pretreated with the SFK inhibitor PP2 (500 nM) before 7α,25-OHC stimulation. Data are presented as mean ± SD (n = 3 biological replicates). Statistical significance was determined by two-way ANOVA followed by Tukey's post hoc test. *$P < 0.05$, **$P < 0.01$, ***$P < 0.001$, ****$P < 0.0001$; ns, not significant.

Figure S8.

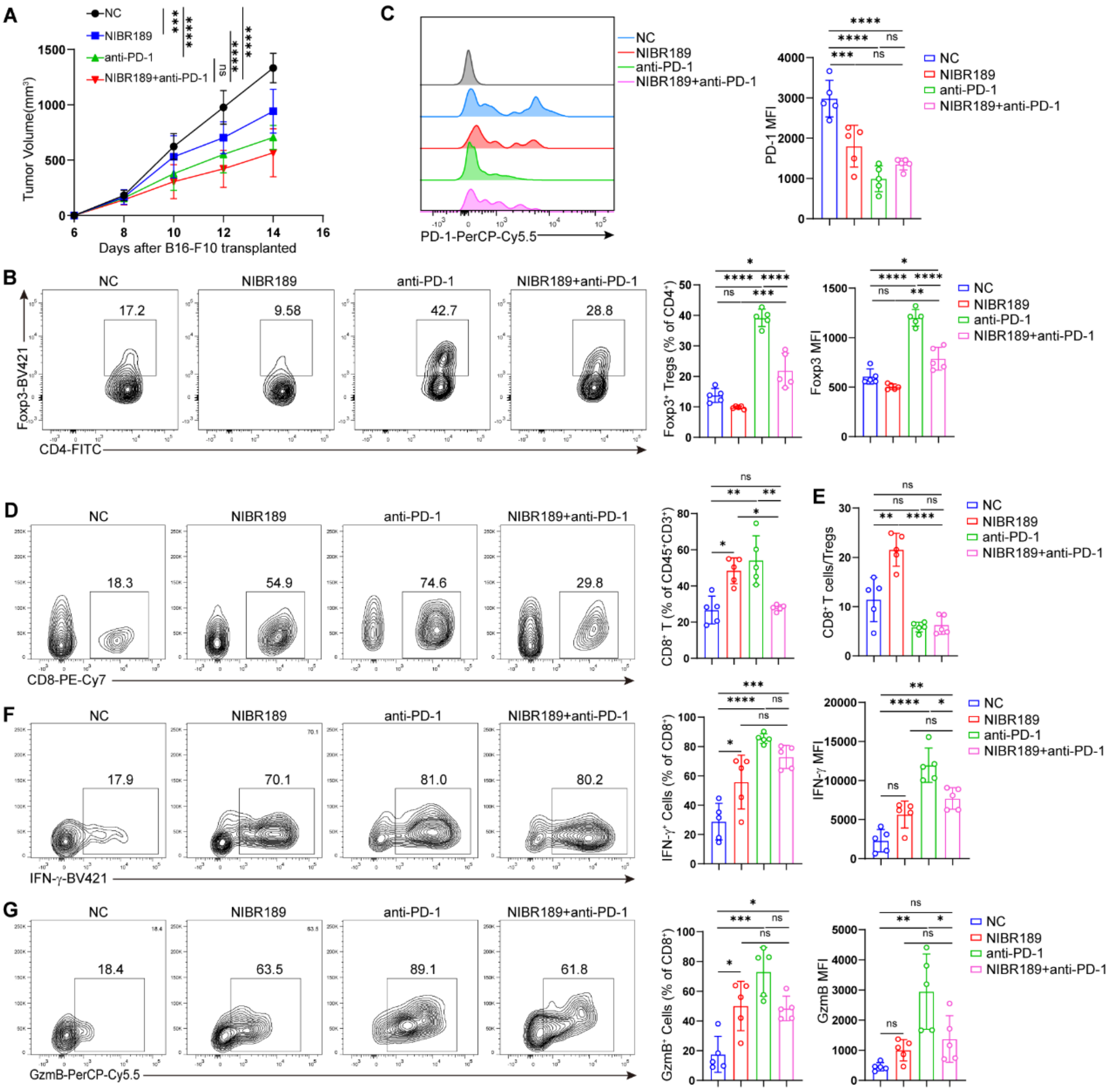

Figure S8. GPR183 blockade remodels the immune landscape under anti-PD-1 therapy in the B16F10 melanoma model. (A) B16F10 tumor growth curves in mice fed a high-cholesterol diet (HCD) and treated with vehicle (NC, negative control), NIBR189, anti-PD-1, or a combination of NIBR189 and anti-PD-1. (B) Representative flow cytometry plots (left)

and quantification (right) of intratumoral Treg frequencies and Foxp3 MFI across the four treatment groups. (C) Representative flow cytometry histograms (left) and quantification (right) of PD-1 expression (MFI) on intratumoral Tregs. (D) Representative flow cytometry plots (left) and quantification (right) of tumor-infiltrating $CD8^+$ T cell frequencies. (E) Statistical quantification of the intratumoral $CD8^+$ T cell/Treg ratio across the indicated groups. (F, G) Representative flow cytometry plots and quantification (frequency and MFI) of effector molecules IFN-γ (F) and GzmB (G) in intratumoral $CD8^+$ T cells. Data in (A–G) are presented as mean ± SD (n = 5 biological replicates, mice per group). Statistical significance was determined by two-way ANOVA followed by Tukey's post hoc test. *$P < 0.05$, **$P < 0.01$, ***$P < 0.001$, ****$P < 0.0001$; ns, not significant.

Tables

Table S1. Association of CH25H$^+$CYP7B1$^+$ stromal cells with clinicopathologic characteristics in gastric cancer patients.

Characteristic	No. of patients	CH25H$^+$CYP7B1$^+$ cells (% of Vimentin$^+$ stromal cells)	*P* value
**Age (years)**			**0.372**
＜60	48	25.9 ± 10.2	
≥60	45	24.1 ± 9.5	
**Gender**			**0.645**
Male	64	24.7 ± 9.7	
Female	29	25.8 ± 10.3	
**TNM stage**			**<0.001***
I–II	18	17.5 ± 6.2	
III–IV	75	26.9 ± 9.8	
**N stage**			**0.005***
N0	17	18.2 ± 6.5	
N1	17	23.5 ± 10.6	
N2	29	26.9 ± 7.9	
N3	30	28.0 ± 11.1	

Note: Vimentin was used as a marker for stromal cells. The percentage of CH25H$^+$CYP7B1$^+$ cells was calculated relative to the number of Vimentin$^+$ stromal cells.

Table S2. Association of GPR183^{+}Foxp3^{+} Treg frequency with clinicopathologic characteristics in gastric cancer patients.

**Characteristic**	**No. of patients**	**GPR183^{+}Foxp3^{+} cells (% of CD4^{+} cells)**	***P* value**
**Age (years)**			**0.141**
＜60	48	25.9 ± 9.5	
≥60	45	22.8 ± 10.8	
**Gender**			**0.974**
Male	64	24.4 ± 9.9	
Female	29	24.4 ± 11.1	
**TNM stage**			**0.011***
I–II	18	18.9 ± 4.9	
III–IV	75	25.7 ± 10.7	
**N stage**			**0.137**
N0	17	19.8 ± 5.3	
N1	17	27.8 ± 12.2	
N2	29	25.0 ± 11.8	
N3	30	24.4 ± 8.8	

Note: The frequency of GPR183^{+}Foxp3^{+} Tregs was calculated relative to the total number of CD4^{+} T cells within tumor tissues.

Table S3. Antibodies Used in Multiplex Immunofluorescence

Target	Supplier	Catalog No.	Dilution
CD4	CST	48274	1:800
Foxp3	CST	98377	1:100
GPR183	Invitrogen	PA5-100950	1:500
Vimentin	Abclonal	A19067	1:900
CH25H	Bioss	bs-23620R	1:250
CYP7B1	Proteintech	24889-1-AP	1:700

Table S4. Primers used for Genotyping

Target Allele	Primer Name	Sequence (5' to 3')	Expected Band Size (bp)
Gpr183$^{-/-}$	Wild type Forward	ATCTAGCACCAGGCACAG	WT: 659 bp
	Wild type Reverse	AAGTCAGGTTCAAAGGGA	
	Mutant Forward	GGCTTCTCCACTTTCACT	Mut: 715 bp
	Mutant Reverse	CAGCATTTCTACCGTTGT	
Gpr183$^{fl/fl}$	Forward	TGCAAAGGGTATAAGAGAAAGGTCA	WT: 177 bp<br>Mut: 211 bp
	Reverse	TGTGCTGTGAAGTCCCAAGTG	
Foxp3^{Cre}	Wild type Forward	CCCTTGACCTCAAAACCAAG	WT: 408 bp
	Mutant Forward	TGGCTGGACCAATGTGAAC	Mut: 511 bp
	Common Reverse	GTGTGACTGCATGACTAACTTTGA	
Cd4^{Cre}	Wild type Forward	GTTCTTTGTATATATTGAATGTTAGCC	WT: 153 bp
	Wild type Reverse	TATGCTCTAAGGACAAGAATTGACA	
	Mutant Forward	TTGAACCCCAACTAGAGTCGAA	Mut: 248 bp
	Mutant Reverse	AAGCTCTATAAGGCAAAGGGTACAGTC	

Table S5. Cell Isolation and Specialized Flow Cytometry Kits

Reagent	Supplier	Catalog No.
Naïve $CD4^+$ T Cell Isolation Kit II, human	Miltenyi Biotec	130-094-131
Naïve $CD4^+$ T Cell Isolation Kit, mouse	Miltenyi Biotec	130-104-453
$CD4^+CD25^+$ Regulatory T Cell Isolation Kit, mouse	Miltenyi Biotec	130-091-041
MojoSort™ Mouse CD4 Naïve T Cell Isolation Kit	BioLegend	480040
True-Nuclear™ Transcription Factor Buffer Set	BioLegend	424401
Foxp3/Transcription Factor Staining Buffer Set	eBioscience	00-5523-00
BD Phosflow™ Fix Buffer I	BD Biosciences	557870
BD Phosflow™ Perm Buffer III	BD Biosciences	558050
Flow Cytometry Staining Buffer	BD Biosciences	00-4222-57
True-Phos™ Perm Buffer	BioLegend	425401
Fixation Buffer	BioLegend	420801
Cell Staining Buffer	BioLegend	420201

Table S6. Cytokines and T Cell Activation Reagents

Reagent	Supplier	Catalog No.
Recombinant human IL-2	Abclonal	RP01039
IL-2 protein, mouse	MCE	HY-P7077
TGF beta 1, Human	MCE	HY-P7118
TGF beta 1, Mouse/Rat	MCE	HY-P70648
Anti-mouse CD3ε Antibody	BioLegend	100340
Anti-mouse CD28 Antibody	BioLegend	102116
Dynabeads Human T-Activator CD3/CD28	Gibco	11131D

Table S7. Chemical Reagents and Small Molecules

Reagent	Supplier	Catalog No.	Purity
7α,25-OHC	MCE	HY-113962	≥99.88%
NIBR189	MCE	HY-12336	≥99.71%
Bemcentinib	MCE	HY-15150	≥99.92%

Table S8. Antibody Used for In Vivo Therapy

Reagent	Clone	Supplier	Catalog No.	Dose
In vivo anti-mouse PD-1 Recombinant mAb	RMP1-14	STARTER	S0B0594	10 mg/kg

Table S9. Antibodies Used in Flow Cytometry and Intracellular Staining

Reagent	Fluorochrome	Clone	Supplier	Catalog No.
CD45	APC-Cy7	30-F11	BioLegend	103116
CD3	APC	17A2	BioLegend	100236
CD4	FITC	A161A1	BioLegend	357406
CD4	FITC	GK1.5	BioLegend	100406
CD4	BV421	RM4-4	BioLegend	116023
Foxp3	AF647	206D	BioLegend	320114
Foxp3	BV421	MF-14	BioLegend	126419
Foxp3	PE	MF-14	BioLegend	126404
Foxp3	AF488	MF-14	BioLegend	126406
CD45.1	APC-Cy7	A20	BioLegend	110716
CD45.2	APC	104	BioLegend	109814
PD-1	PerCP-Cy5.5	RMP1-30	BioLegend	109104
CTLA-4	PE-Cy7	UC10-4B9	BioLegend	106314
IL-10	PE-Cy7	JES5-16E3	BioLegend	505026
LAP	PerCP-Cy5.5	TW7-16B4	BioLegend	141410
CD8	PE-Cy7	53-6.7	BioLegend	100722
IFN-γ	BV421	XMG1.2	BioLegend	505830
Granzyme B	PerCP-Cy5.5	QA16A02	BioLegend	372212
CCR7	PE	4B12	BioLegend	120106
CD62L	BV421	MEL-14	BioLegend	104435
CD69	PE	H1.2F3	BioLegend	104508
CD103	BV421	2E7	BioLegend	121421

Ki-67	BV421	16A8	BioLegend	652411
Cleaved caspase 3	PE	13C12	Bioss	bsm-61090R
Axl	APC	MAXL8DS	Invitrogen	17-1084-82
p-Axl	APC	Polyclonal	Bioss	bs-5181R
p-Axl	BV421	Polyclonal	Bioss	bs-5181R
p-Axl	PerCP-Cy5.5	Polyclonal	Bioss	bs-5181R
p-JAK2	PE-Cy7	Polyclonal	Bioss	bs-2485R
p-JAK2	PerCP-Cy5.5	Polyclonal	Bioss	bs-2485R
p-STAT5	FITC	SRBCZX	Invitrogen	11-9010-42
p-STAT5	PE	SRBCZX	Invitrogen	12-9010-42
CellTrace Violet	BV421		Invitrogen	C34557
Zombie	BV510		BioLegend	423102
TruStain FcX™ PLUS			BioLegend	156604
